# Bisphenols-enhanced platelet aggregation via TP, P2Y_12_, PAR1 and PAR4 receptors: a thrombotic legacy in a plastic-ubiquitous world

**DOI:** 10.3389/fphar.2026.1840963

**Published:** 2026-06-22

**Authors:** Opata Edward Kwame, Aurora de la Peña-Díaz, Yesenia I. Martínez-Jiménez, Mirthala Flores-García, Emma S. Calderón-Aranda, Irene Lee-Rivera, Rocío Gómez

**Affiliations:** 1 Departamento de Toxicología, Centro de Investigación y de Estudios Avanzados del Instituto Politécnico Nacional, Ciudad de México, México; 2 Departamento de Farmacología, Laboratorio de Trombosis y Fibrinólisis, Facultad de Medicina, Unidad de Investigación UNAM-INC, UNAM, Unidad Foránea, Instituto Nacional de Cardiología “Ignacio Chávez”, Ciudad de México, México; 3 Departamento de Biología Molecular, Instituto Nacional de Cardiología “Ignacio Chávez”, Ciudad de México, México; 4 Departamento de Neuropatología Molecular, Instituto de Fisiología Celular, Universidad Nacional Autónoma de México, Ciudad de México, México

**Keywords:** bisphenols, cardiotoxicity, cardiovascular diseases, haemostasia, microplastics, platelet aggregation, thrombosis

## Abstract

**Background:**

Cardiovascular diseases remain an urgent global health concern addressed by multiple clinical guidelines. Atherothrombosis is characterised by thrombus formation following disruption of atherosclerotic plaque, leading to major adverse cardiovascular events, such as myocardial infarction and stroke. Platelet hyperactivity is a key driver of these outcomes, leading causes of morbidity and mortality worldwide. Environmental xenobiotic exposure has been documented to contribute to atherosclerosis, endothelial dysfunction, and hypertension; however, its effects on thrombosis remain scarcely documented. In the present study, the effects of bisphenol A (BPA) and its most commonly used structural analogues on platelet aggregation were assessed using an *ex vivo* model. *In silico* analyses were also performed to identify potential receptors through which bisphenols may induce platelet aggregation. Additionally, our findings were contextualised within a systematic review spanning 10 years.

**Methods:**

Platelet-rich plasma from healthy middle-aged men was incubated (30 min at 37°) with different bisphenols at concentrations ranging from 5 pM to 500 nM. Following incubation, platelet aggregation was induced with adenosine diphosphate (ADP) and measured with a Lumi-Aggregometer. Results were presented as standardised mean differences (SMD). A systematic review spanning 10 years was conducted to compare our findings. To explain our results, *in silico* analyses were conducted to evaluate the binding affinity and interaction models of bisphenols at platelet receptors relative to their reference agonists and antagonists. Receptors were selected based on the structural similarity between their cognate agonist and bisphenols. Binding affinity was assessed for the thromboxane A_2_ (TP), the purinergic P2Y-subtype-12 (P2RY_12_) and the protease-activated receptors 1 (PAR1) and 4 (PAR4).

**Results:**

Bisphenols did not provoke platelet aggregation spontaneously. However, once ADP was added, they induced a strong and prolonged platelet aggregation (SMD: 1.90; _95%_ CI: 1.68–2.11) in the *ex vivo* model, with a potency ranking of BPF > TDF > BPS > BPA > BPAF. These findings were reinforced by the results from other studies in which human platelets were exposed *ex vivo* to bisphenols. The *in silico* analyses revealed that bisphenols exhibit favourable binding affinities for TP, which may explain the *ex vivo* findings. Bisphenols also showed binding affinity for P2Y_12_, PAR1, and PAR4.

**Conclusion:**

Our study demonstrated that exposure to bisphenols enhanced and prolonged ADP-induced platelet aggregation and, for the first time, illuminated their interactions with key receptors involved in this complex process. Nonetheless, bisphenols may also exert other pleiotropic effects that further contribute to platelet aggregation. These results highlight the remarkable toxicity of these xenobiotics and their significant impact on haemostatic balance.

## 1 Introduction

Haemostasis and thrombosis are “yin” (physiological) and “yang” (pathological) complex processes, where procoagulant and anticoagulant mechanisms are harmoniously regulated. Haemostasis responds to vascular injury by forming a temporary clot; platelets, coagulation proteins, and the vascular endothelium interact coordinately to maintain vascular integrity (Kenet et al., 2026). Thrombosis, by contrast, leads to partial or complete obstruction of blood flow in a damaged blood vessel, forming a thrombus (Aslan, 2026). These two processes are modulated by platelets, dynamic and highly sensitive cells, that carry a diverse repertoire of specialised receptors activated by their agonists.

To maintain vascular integrity, platelets need to stop bleeding and form a stable haemostatic thrombus, following three coordinated steps: 1) adhesion, 2) activation, and 3) aggregation (Denorme, 2026). In the first step, platelets adhere to collagen, fibronectin, laminin, and von Willebrand factor (vWF) from the subendothelial matrix, which is exposed following vascular injury (Kenet et al., 2026). This adherence is mediated by platelet adhesion receptors, such as glycoprotein complexes (GP-Ib-IX-V), which bind to collagen-bound vWF, favouring further interactions as platelets roll along the vessel wall. From such interactions, platelets slow down and engage GPVI and integrin α2β1, which in turn, bind to collagen, triggering signal transduction that induces cytoskeletal rearrangements and shape changes, activating platelets. This step is amplified by increased intracellular calcium (Ca^2+^) levels, releasing platelet granule-derived factors, such as adenosine 5′-diphosphate (ADP) and thromboxane A2 (TxA2); as well as thrombin (from the coagulation cascade), which stimulates G protein-coupled receptors (GPCRs). P2Y-subtype-12 (P2RY_12_) receptor binds to ADP, and the thromboxane A_2_ receptor (TP) binds to TxA2. Fibrinogen, vWF, and P-selectin (adhesion protein) and procoagulant mediators are also released, whereas integrins shift to a high-affinity state (Denorme, 2026). Once platelets are activated, they further aggregate through the binding of αIIbβ3 to fibrinogen and vWF. Thrombin, the most potent endogenous platelet activator, binds to protease-activated receptors 1 and 4 (PAR1, PAR4) and converts fibrinogen to fibrin, which stabilises the platelet plug (Denorme, 2026). Apart from increasing platelet aggregation, P-selectin promotes inflammation by recruiting immune cells, a pathognomonic signature of endothelial dysfunction (Palacios-Valladares et al., 2024). If platelet aggregation is excessive, it leads to thrombosis and its clinical manifestations: myocardial infarction (MI) and stroke.

Thrombosis accounts for approximately one-quarter of all deaths globally. MI, stroke, and venous thromboembolism are the principal acute manifestations of cardiovascular diseases (CVDs) and are associated with atrial fibrillation, coronary artery disease, and heart failure (de Vecchi et al., 2026). CVDs are the leading cause of morbidity and mortality worldwide. These multifactorial diseases are initiated by atherosclerosis, a primary pathological process characterised by lipid infiltration, endothelial injury, inflammation, and plaque formation (Dabravolski et al., 2025). However, the underlying cause of MI and stroke conditions is atherothrombosis (Huse et al., 2026). Atherothrombosis is a chronic vascular condition triggered by highly modified components of atherosclerotic plaque that are exposed following its rupture or erosion, leading to platelet activation and thrombosis (von Hundelshausen et al., 2026).

Family history, pre-existing comorbidities (i.e., diabetes, hyperlipidaemia, hypertension, obesity, etc.), sedentary lifestyle, alongside alcohol and tobacco dependencies, increase atherothrombotic risk (Camacho-Mejorado et al., 2020). Chemical pollutants, such as heavy metals, pesticides, polycyclic aromatic hydrocarbons and plastics, negatively impacted cardiovascular health. Bisphenol A (BPA), an endocrine-disrupting chemical (EDC) with ubiquitous distribution worldwide, has been associated with endothelial dysfunction, hypertension, lipid metabolism alterations, and consequently with atherosclerotic plaque formation; a cornerstone of CVDs. Structural analogues that preserve its main features, such as durability, lightness, low cost, strength, transparency, and versatility, are increasingly being used (Palacios-Valladares et al., 2024). Although xenobiotic exposure on CVDs has been widely studied, its effect on thrombosis has received little attention. *In vivo* studies have demonstrated that BPA and its structural analogues, including bisphenol S (BPS) and bisphenol AP, promote thrombosis (Chagas et al., 2021; Akhter et al., 2023; Li et al., 2023; Mladenka et al., 2025). *Ex vivo* human studies have likewise demonstrated the effects of BPA, BPS, and related alternatives on platelet aggregation and haemostasis (Chagas et al., 2021; Burgos et al., 2025; Hrubsa et al., 2025; Hrubsa et al., 2026). Other structural analogues of BPA, such as bisphenols F, BPS and AF, have received little attention, particularly regarding their role as haemostatic disruptors. In the present study, the effects of BPA and its most used structural analogues on platelet aggregation were assessed through an *ex vivo* model. *In silico* analyses were also performed to identify potential receptors through which bisphenols may induce platelet aggregation. Additionally, our findings were compared with several studies from a systematic review spanning 10 years, to corroborate the enhanced and prolonged ADP-induced platelet aggregation observed in the present study.

## 2 Materials and methods

### 2.1 Human platelet preparation

The Ethics Committee of the Medicine School of the National Autonomous Mexican University (UNAM) approved the protocol and sample collection (FD/DI/055/2022). All individuals signed an informed consent form. This study agrees with the principles established by the Declaration of Helsinki.

Venous blood was obtained from healthy Mexican Mestizo male donors (age range: 18–55 years) who had previously met the requirements outlined in the Official Mexican Standard (acronym in Spanish, NOM) for collecting human blood and its components for therapeutic purposes (NOM-253-SSA1-2012). All these individuals were obtained from the blood bank of the *Instituto Nacional de Cardiología* “Ignacio Chávez”. These donors did not present CVD’s comorbidities at the time of sample collection (i.e., obesity and type 2 diabetes, T2D), nor were they smokers. Obesity was defined as a body mass index ≥30 kg/m^2^, whereas T2D was defined as glycosylated haemoglobin levels >5.70% and fasting glucose >100 mg/dL (5.60 mmol/L). None of these donors used anticoagulants, antifibrinolytics, antithyroid, antihistaminic, anti-inflammatory, or antioxidant supplementation during the 2 weeks preceding the sample collection. The participants did not smoke or drink alcohol for at least 24 h before the sample collection.

All samples were drawn using a 19G needle into sodium citrate tubes (0.109 M, in a 9:1 ratio of blood to anticoagulant). Blood samples were centrifuged at room temperature (20 °C–24 °C), first at 60 x g for 2 min to obtain platelet-rich plasma (PRP), followed by a second cycle at 900 x g for 20 min to obtain platelet-poor plasma (PPP). Platelet concentration in PRP samples was adjusted to 250 × 10^3^ platelets/μL using PPP with a Cell-Dyn Ruby haematology analyser (Abbot, NJ, United States). All assays were conducted within 2 h of sample collection to ensure platelet viability.

### 2.2 Chemicals

Bisphenol A, its structural analogues, and their parent compound (4,4′-thiodiphenol, TDP), were purchased from Sigma-Aldrich (St. Louis, MO, United States) with the following Chemical Abstract Service (CAS) numbers and purity: BPA (CAS no. 80-05-7, purity >99%), BPF (CAS no. 620-92-8, purity >98%), BPS (CAS no. 80-09-1, purity >98%), BPAF (CAS no. 1478-61-1, purity >99%), and TDP (CAS no. 2664-63-3, purity >99%). Such compounds were dissolved in dimethyl sulfoxide (DMSO), which was also purchased from Sigma-Aldrich (St. Louis, MO, United States).

### 2.3 Preliminary analyses

#### 2.3.1 Effect of DMSO on platelet aggregation

Prior to conducting the experiments, the effect of the vehicle in which bisphenols were dissolved on platelet aggregation was assessed. Thus, the effect of five DMSO concentrations (0.0001%, C1; 0.001%, C2; 0.01%, C3; 0.1%, C4 and 1%, C5) on platelet aggregation was examined. Sex-related differences in platelet reactivity, with women exhibiting a higher platelet response to certain agonists such as adenosine diphosphate (ADP) and collagen, have been documented (Gasecka et al., 2023). Furthermore, menstrual cycle phase and menopausal status, endogenous sex hormone fluctuations, hormone replacement therapy and oral contraceptive use, introduce additional variability, particularly when EDCs such as bisphenols are being tested. Accordingly, PRP pools from healthy male donors, with five individuals per pool, were used. These experiments were conducted using four agonists: 1) ADP (cat # 384), 2) collagen (cat # 385), 3) epinephrine (cat # 393), and 4) thrombin (cat # 386), all purchased from Chrono-PAR Corporation (Havertown, PA, United States). Human platelets were incubated for 30 min at 37 °C with the different DMSO concentrations tested. Each experiment was conducted, independently, at least trice. Following exposure, samples were stimulated, separately, with each agonist under the conditions previously established in our laboratory: ADP [5 μM], collagen [2 μg/mL], epinephrine [1.1 μM], and thrombin [1 unit]. Platelet aggregation in the control group (CTL), in which phosphate-buffered saline (PBS) was used, was set to 100%. Platelet aggregation (% over 6 min) was monitored using a Lumi-Aggregometer Model 560CA (Chrono-Log, Havertown, PA, United States).

#### 2.3.2 Effect of bisphenols—*per se*—on platelet aggregation

The effect of all bisphenols and their parent compound on platelet aggregation, in the absence of any agonist, was assessed at 3.5 nM; the lowest BPA environmental concentrations reported in human matrices (Sajiki et al., 1999). These experiments were conducted under the conditions described above, including the platelet induction using the four agonists. All bisphenols and their parent compound were dissolved to a final DMSO concentration of 1% v/v. PRP without and with agonists served as negative and positive controls, respectively. Platelet aggregation (% over 6 min) was monitored using a Lumi-Aggregometer Model 560CA (Chrono-Log, Havertown, PA, United States).

#### 2.3.3 Generation of concentration-response curves

Concentration-response curves were generated, using ADP [5 μM] as the sole inducer, to derive the half-maximal effective concentration (EC_50_) and to identify the concentration range over which these compounds modify ADP-induced platelet aggregation. Multiples of 3.5 nM were used as the bases for the tested concentrations (Sajiki et al., 1999). Six nanomolar concentrations (0.00035, 0.035, 0.35 3.5, 35, and 350 nM) were examined with BPA (the most widely used bisphenol) and BPF (the second-most commonly detected bisphenol in human matrices) (Duan et al., 2026). Bisphenols were dissolved to a final DMSO concentration of 1% v/v. Each experiment was conducted, independently, three times using platelet pools (five individuals per pool) from healthy male donors. Platelet aggregation (% over 6 min) was monitored using a Lumi-Aggregometer Model 560CA (Chrono-Log, Havertown, PA, United States). The results from these experiments enabled identification of the concentration range over which the compounds modified ADP-induced platelet aggregation. Based on these results, 4 nM bisphenol concentrations were selected (0.005, T1; 0.232, T2; 10.80, T3; and 500 nM, T4), which were subsequently applied to each bisphenol and its parent compound in all subsequent experiments.

### 2.4 Treatment conditions

Given the individual variability, each experiment was conducted in PRP pools with five individuals per pool (range: 5 to 8 pools per treatment). These pools were exposed to the different nM concentrations mentioned before and incubated at 37 °C for 30 min. PRP with 1% DMSO in phosphate-buffered saline (PBS) was used as the control group (CTL). Each experiment was conducted at least nine times (range: 9–13; mean = 10.60) independently. Following exposure, samples were stimulated with ADP (5 µM). The concentration-response curves were generated using a Lumi-Aggregometer Model 560CA (Chrono-Log, Havertown, PA, United States) to monitor platelet aggregation (% for 6 min). Platelet aggregation observed in the CTL was regarded as 100%; the bisphenols’ response was expressed as a percentage relative rate.

### 2.5 Statistical analyses

Normality was assessed using the Shapiro-Wilk test and other tests with the Moments v0.14.1 and nortest v1.0.4 libraries (Gross, 2015; Komsta, 2022). Homoscedasticity was evaluated using the Fligner-Killeen test, followed by a one-way analysis of variance (ANOVA). As this test was not-significant, Welch’s test followed by Dunnett’s *post hoc* test was performed. These analyses were carried out using the libraries HH v3.1.53, rstatix v0.7.2, and DescTools v0.99.60 (Heiberger, 2015; Kassambara, 2023; Signorell, 2025). Dunnett’s *post hoc* test was presented as the mean ± standard error (SE). For the analysis of EC_50_ curves, the libraries dcr v.3.0-1and scales v1.4.0 were used (Ritz et al., 2015; Wickham and Seidel, 2025). All statistical analyses were conducted in the R language; a *p-*value ≤0.05 was considered statistically significant. Plots were conducted in the tidyverse v2.00 library (Wickham et al., 2019). Script with the conducted analyses is available in the Supplementary Information.

### 2.6 Bioinformatic analyses

Bioinformatic analyses were performed to evaluate the binding affinity and interaction models of bisphenols to platelet receptors relative to their reference agonist and antagonist. The target receptors assessed were P2RY_12_, TP, PAR1 and PAR4. The endogenous ligands for these receptors were ADP in the case of P2RY_12_, TxA_2_ for TP, and thrombin for PAR1 and PAR4 (Coughlin, 2000; Cattaneo, 2015; Yan et al., 2016). For this purpose, protein-ligand docking analyses were performed with AutoDock Vina 1.2.0 and SwissDock (Eberhardt et al., 2021; Bugnon et al., 2024). To explore the molecular interactions between PAR1 and PAR4 with thrombin, protein-protein docking simulations were conducted using the LZerD and MDockPP web servers (Huang and Zou, 2010; Christoffer et al., 2021). The binding energy values (kcal/mol) from the resulting complexes were obtained with the PRODIGY server (Xue et al., 2016).

#### 2.6.1 Ligand preparation and protein-ligand docking

Crystallographic structures for P2RY_12_, TP, PAR1, and Thrombin were retrieved from the Protein Data Bank (PDB; https://www.rcsb.org/) under the PDB IDs: 4PXZ, 6IIU, 3VW7, and 1PPB, respectively (Bode et al., 1989; Zhang et al., 2012; Zhang et al., 2014; Fan et al., 2019). Water molecules and non-essential heteroatoms were removed to prepare the structures, followed by the addition of polar hydrogens and the assignment of Gasteiger partial charges with AutoDock Tools (Eberhardt et al., 2021).

Given that the PAR4 crystallographic structure is currently unavailable, the three-dimensional model was generated using Swiss-Model from the FASTA sequence (UniProt ID: Q96RI0) (Waterhouse et al., 2018). The Swiss-Model platform was chosen over a direct AlphaFold prediction because it enables a template-based approach that integrates specific structural refinement with standardised validation metrics (Terwilliger et al., 2022). Thus, the modelling process utilised AlphaFold-derived structures as templates, leveraging their high-accuracy coordinates while ensuring the final model met rigorous quality criteria within the Swiss-Model pipeline. The resulting model’s structural quality was evaluated using the Global Model Quality Estimate (GMQE), the MolProbity score, and the Ramachandran plot with the Swiss-Model Server (Schwede et al., 2003). GMQE values close to 1 indicated high model reliability; values below 0.50 indicated moderate to low confidence (Waterhouse et al., 2018). Values below 2.00 were considered acceptable for MolProbity; models with values ≥90% of residues favouring regions were considered structurally reliable in the Ramachandran plot (Wlodawer, 2017).

The three-dimensional structures of the bisphenols, agonist and antagonist, were obtained from the PubChem database (https://pubchem.ncbi.nlm.nih.gov/), with the following specific identifiers: BPA (ID: 6,623), BPF (ID: 12,111), BPS (ID: 6,626), BPAF (ID: 73,864), and TDP (ID: 17,570), ADP (DB: 16,833), TxA2 (ID: 5,280,497), Cangrelor (ID: 9,854,012), Ramatroban (ID: 123,879), Vorapaxar (ID: 10,077,130), and Bms-986120 (ID: 72,190,270). In all these structures, hydrogen atoms were added, and protonation states were assigned to match physiological pH (7.40) using the Dock Prep tool in UCSF Chimera (Huang et al., 2014). Side-chain orientations were also optimised, and all non-functional heteroatoms, including residual solvent molecules and crystallographic additives, were removed to ensure a clean starting structure. Gasteiger charges were subsequently computed, and polar atoms were retained using AutoDock Tools (Morris et al., 2009).

Based on the co-crystallised ligand coordinates in each structure, the docking search boxes were defined for use in AutoDock Vina 1.2.0 (Eberhardt et al., 2021). These analyses were carried out with an exhaustiveness value of 8, yielding 9 possible conformations per ligand. The resulting coordinates were compared with PAR1’s active site, which served as a reference structure. The docking boxes’ dimensions and the centre coordinates for each receptor were presented in the Supplementary Table S1.

To validate the methodology and ensure an accurate definition of the binding site, redocking was performed using the co-crystallised ligands from the aforementioned PDB structures (4PXZ, 6IIU, 3VW7, and 1PPB) (Bode et al., 1989; Zhang et al., 2012; Zhang et al., 2014; Fan et al., 2019). This validation was achieved by calculating the Root Mean Square Deviation (RMSD) between the predicted and experimental ligand poses; an RMSD value ≤2.0 Å indicated that the ligand pose was “near-native”. The attracting cavities option for the automatic potential exploration alternative binding sites was used in SwissDock (Grosdidier et al., 2011). The protein-ligand resulting complexes were visualised in PyMOL, Discovery Studio Visualiser, and PDBsum (Yuan et al., 2017; Laskowski et al., 2018; Umi Baroroh et al., 2023). The resulting complexes were focused on the binding energy values (kcal/mol), and key molecular interactions (e.g., hydrogen bonds, hydrophobic contacts, and π–π stacking).

#### 2.6.2 Protein-protein docking

To explore the molecular interactions between PAR1 and PAR4 and their effector protein, thrombin (PDB ID: 1PPB), protein-protein docking simulations were performed (Bode et al., 1989). For each receptor-thrombin pair, the 3D structures were uploaded in PDB format to the LZerD and MDockPP platforms. For the LZerD platform, residue-based restraints were defined according to the known catalytic and interaction sites reported in the literature for PAR1-thrombin and PAR4-thrombin complexes (Nieman and Schmaier, 2007; Gandhi et al., 2011; Christoffer et al., 2021). The resulting docked complexes were ranked according to the Z-score and cluster density criteria. Similarly, docking in MDockPP was performed for the same PAR1-thrombin and PAR4-thrombin pairs, and the best-ranked models were selected based on the ITScorePP energy function. Finally, the top-ranked complexes obtained from both servers were further analysed using the PRODIGY web server to estimate the binding free energy (ΔG°, in kcal/mol) and the number of interfacial contacts between residues of the interacting proteins (Xue et al., 2016).

### 2.7 Systematic review and meta-analyses

To reinforce the results, our findings was compared to analogous published data. Thus, a systematic review was conducted following the Preferred Reporting Items for Systematic Reviews and Meta-Analyses 2020 (PRISMA) statement (Page et al., 2021). The inclusion criteria were restricted to articles published in English and Spanish that reported platelet aggregation determined in the presence (exposure group) and absence (control group) of bisphenol, from *ex vivo*, *in vitro,* or *in vivo* studies. Five databases (e.g., Cochrane library, Epistemonikos, Lilacs, PubMed, and Scopus) were consulted. Articles published from January 2016 to January 2026 were reviewed. The search strategy included the Medical Subject Headings (MeSH) terms “aggregation, platelet”, “blood clot”, “coagulation, blood”, and “thrombosis”. These headings were combined with the term “bisphenol compounds”. Four reviewers independently screened all studies retrieved from the search strategy, using title and/or abstract as eligibility criteria. All these reviewers conducted full-text reviews of potential eligibility documents. They evaluated the quality, internal validity, and risk of bias using SYRCLE’s bias tool for *in vivo* studies and the AMSTAR 2 tool for human studies (Hooijmans et al., 2014; Shea et al., 2017). Only studies considered low risk of bias by all reviewers were included; disputes were resolved through group discussions.

Commentaries, newsletters, reviews, overviews, meta-analyses and overlapping publications were excluded. Data extraction included the first author’s name, publication year, country, study type, study model, sample size, mean and SD for exposed and non-exposed groups, and xenobiotics. Other data were obtained from the manuscripts (tables and figures) and supplementary information, including platelet aggregation (%), prothrombin time (PT) in International Normalised Ratio (INR) units, and activated partial thromboplastin time (aPTT) in seconds. *In vivo* models, bleeding time, blood clotting time, and fibrinogen were also obtained. In the case of the data obtained from figures, two reviewers independently approximated these data, which were reported as mean ± SD (Supplementary Table S1). Platelet aggregation was reported as a percentage relative to the vehicle control, which was set at 100%. Meta-analyses were performed using the R library metafor v4.8-0 (Viechtbauer, 2010). The effect size (expressed in standard deviation, SD units) was presented as the standardised mean difference (SMD) between the CTL and the exposed group, along with its 95% confidence interval (_95%_ CI). The Hedges’ *g* correction—SMD*g*—was applied to reduce bias, as most studies used small samples, and results were represented in a forest plots (Lin and Aloe, 2021). The effect size with respect to CTL was considered as small (SMD*g* ≤ 0.20), medium (SMD*g* ≥ 0.50) and large (SMD*g* ≥ 0.80), according to the conventional criteria (Hedges, 1985).

Heterogeneity among studies was evaluated using the heterogeneity index (*I* ^2^); values of 25%, 50%, and 75% were classified as low, moderate, and high heterogeneity, respectively. A random-effects model was applied if the *I* ^2^ value exceeded 25%; otherwise, a fixed-effects model was used. To address heterogeneity across studies, subgroup meta-analyses and meta-regression were conducted (Hedges, 1985; Harrer et al., 2019). Influence analyses using the leave-one-out method, the Baujat plot, and the graphic display of heterogeneity (GOSH) plots were also carried out in R using metafor v4.8-0 (Viechtbauer, 2010).

## 3 Results

None of the five DMSO concentrations tested had a significant effect on platelet aggregation compared with the CTL group. Similarly, no differences were found among the effects of the four agonists on platelet aggregation induction (Supplementary Figure S1). Hence, all experiments were conducted using only ADP [5 μM] as the agonist, and all bisphenols were dissolved in a final DMSO concentration of 1% v/v, which provided optimal solubility.

Once it was verified that DMSO did not affect platelet aggregation, *ex vivo* exposure to different bisphenols was assessed. The time-course recordings showed that exposure to bisphenols, *per se*, did not induce platelet aggregation; an agonist was required (Supplementary Figure S2).

The concentration-response assessment showed that bisphenols differed in their ability to enhance ADP-induced platelet aggregation (a compound-specific effect). Moreover, bisphenols did not increase uniformly or linearity across the full concentration range (hormetic patterns). For BPA (EC_50_ = 27.65 nM), the response oscillated between 7% and 12% relative to the CTL. For BPF (EC_50_ = 340 nM), the response ranged between 5% and 20% relative to the CTL. Based on these results, four experimental concentrations (T1-T4) were selected and used in subsequent experiments.

### 3.1 Bisphenols’ exposure enhances platelet aggregation

**FIGURE 1:**
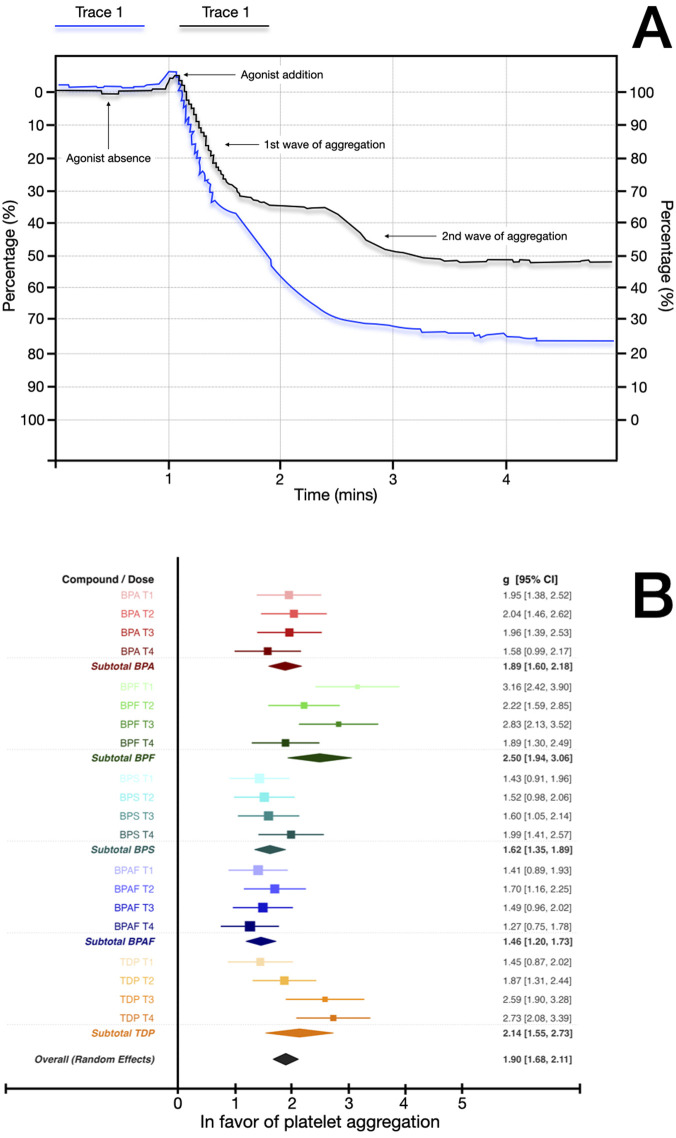
**(A)** Representative curve of the effect of bisphenols on platelet aggregation. **(B)** Pooled and group meta-analyses of the effect of bisphenols on platelet aggregation represented as a forest plot. Footnote: BPA, bisphenol A; BPAF, bisphenol AF; BPF, bisphenol F; BPS, bisphenol S; CTL, control; *I* ^2^, heterogeneity index; TDP, 4,4′-thiodiphenol; T1, 0.005 nM, T2, 0.232 nM; T3, 10.80 nM; T4, 500 nM. The effect size was presented as the standardised mean difference with the Hedges’ g correction along with its 95% confidence interval.

Overall, TDP and bisphenols significantly enhanced and prolonged ADP-induced platelet aggregation, particularly during the secondary aggregation wave, forming an initial peak followed by a small plateau, which was overcome faster than in CTL (Figure 1A). The progression of the trace continued with the initiation of the second platelet aggregation wave, which exhibited an amplified aggregation response, evidenced by the curve’s greater amplitude compared with the CTL. The effect of the four concentrations (T1 – T4) for each tested bisphenol and TDP on platelet aggregation was summarised in a forest plot (Figure 1B). The plot showed that the pooled mean effect across exposure conditions was higher than the CTL mean (SMD*g*: 1.90; 95%CI: 1.68–2.11), indicating an increase in platelet aggregation induced by bisphenols. Exposure to BPF showed the greatest increase (SMD*g*: 2.50; _95%_CI: 1.94–3.06), almost twice that of the CTL group’s mean, even at the lowest concentration. By contrast, BPAF showed the smallest increase (SMD*g*: 1.46; _95%_ CI: 1.20–1.73).

**FIGURE 2:**
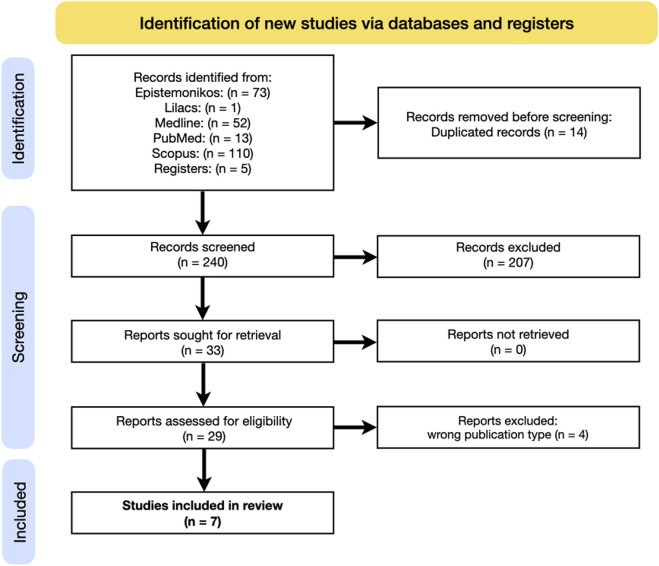
Selection process summary from the systematic review following the PRISMA 2020 statement.

From the records screened from the systematic review (Figure 2), only seven full-text articles (one of which examined both effects in human platelets and in *D. rerio*) documented the procoagulant effects of bisphenols (Supplementary Table S2). Of these, three studies examined the effects of bisphenols using *in vivo* models (Albino and Sprague-Dawley rats and *D. rerio*); data were obtained from only two (*D. rerio* and Albino rats). Nonetheless, these two models were not comparable, and no further analyses were conducted. Of the five studies on *ex vivo* human platelets, data from four were obtained and included in the meta-analyses to explore the overall effect of bisphenols on platelet aggregation. These studies examined the effects of BPA, BPS, and BPA’s new alternatives on platelet aggregation, as well as PT, and aPTT.

**FIGURE 3:**
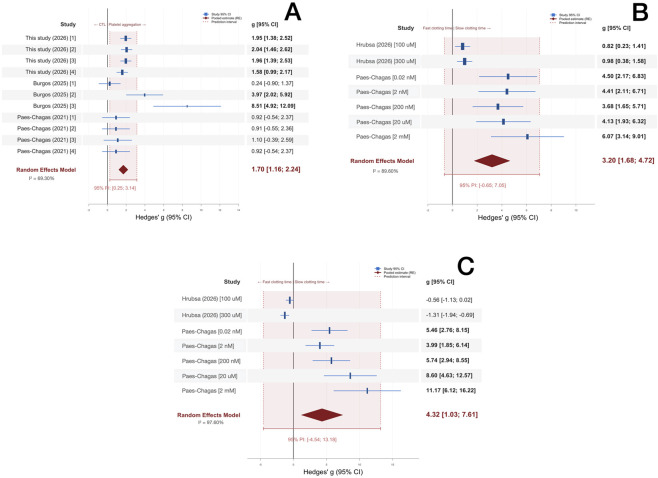
Pooled meta-analysis of the effect of bisphenol A on platelet aggregation, prothrombin time and activated partial thromboplastin time. **(A)** Forest plot of the pooled effect of bisphenol A on platelet aggregation. **(B)** Forest plot of the pooled effect of bisphenol A on prothrombin time. **(C)** Forest plot of the pooled effect of bisphenol A on activated partial thromboplastin time. Footnote: CTL = control; g [95% CI] = standardised mean difference with the Hedge’s g correction along with its 95% confidence interval; *I* ^2^ = Heterogeneity index; 95% PI = Prediction interval with its 95% confidence interval.

Overall, BPA increased the agonist-induced platelet aggregation by at least 1SD (Supplementary Figure S3). Nonetheless, a remarkable heterogeneity among studies was found (*I* ^*2*^ = 98.50%), which persisted even when studies were separated into subgroups. To identify the sources of heterogeneity, meta-regression was carried out (R^2^ = 0.19), revealing different effects on platelet aggregation (Supplementary Table S3): effects induced with ADP (β = 3.83, *p* ≥ 0.05) differed from those induced with Arachidonic Acid (AA; β = −1.12, *p* ≥ 0.05). Influence analyses via a Baujat plot identified the study of Hrubsa et al. (2026), as a potential outlier (Supplementary Figure S3). This finding was reinforced by the leave-one-out analysis, which showed that the exclusion of this article reduced *I* ^*2*^ by 69% (Supplementary Table S3). Once this study was removed the meta-analysis showed a clear contribution of BPA to enhanced platelet aggregation (SMD*g*: 1.70; _95%_ CI: 1.16–2.24) (Figure 3A). Interestingly, PT (SMD*g*_BPA_: 3.20; _95%_CI: 1.68–4.72) and aPTT (SMD*g*_BPA_: 4.32; _95%_CI: 1.03–7.61) showed a concerning adverse effect on these coagulation parameters (Figures 3B,C). A concentration-response pattern was again not found, although low-concentrations appeared to prolong the clotting time, whereas high concentrations appeared to favour the aggregation. However, these patterns should be interpreted with caution, as they are based on only two studies.

**FIGURE 4:**
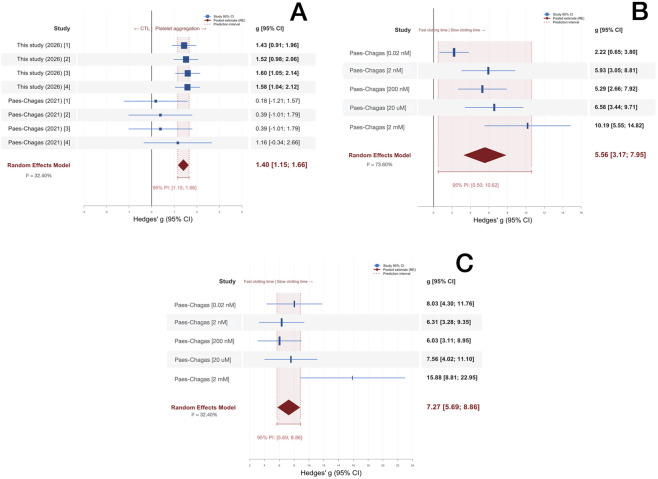
Pooled meta-analysis of the effect of bisphenol S on platelet aggregation, prothrombin time and activated partial thromboplastin time. **(A)** Forest plot of the pooled effect of bisphenol S on platelet aggregation. **(B)** Forest plot of the pooled effect of bisphenol S on prothrombin time. **(C)** Forest plot of the pooled effect of bisphenol S on activated partial thromboplastin time. Footnote: CTL = control; g [95% CI] = standardised mean difference with the Hedges’ g correction along with its 95% confidence interval; *I* ^2^ = Heterogeneity index; 95% PI = Prediction interval with its 95% confidence interval.

The BPS meta-analysis showed, a clear pooled effect enhancing platelet aggregation (SMD*g*: 1.40; _95%_CI: 1.15–1.66), with low to moderate heterogeneity (*I* ^*2*^ = 32.40%). The pooled effect in PT (SMD*g*_BPS_: 5.56; _95%_CI: 3.17–7.95) and aPTT (SMD*g*_BPS_: 7.27; _95%_CI: 5.69–8.86) indicated a prolonged clotting time with BPS (Figure 4). Although a certain effect was found regarding the agonist (ADP β = 1.02, *p* ≤ 0.01 vs*.* AA β = 0.51, *p* ≥ 0.05), both indicated prolonged clotting times.

**FIGURE 5:**
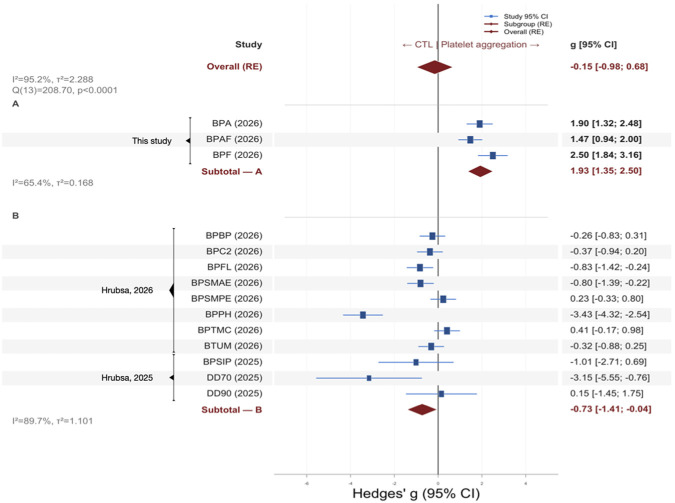
Pooled and group meta-analyses for the effect of bisphenols on platelet aggregation. **(A)** Most commonly used bisphenols. **(B)** New alternative bisphenols. Footnote: BPA, bisphenol A; BPAF, bisphenol AF; BPF, bisphenol F; BPBP, bisphenol BP; BPC2, bisphenol C2; BPFL, bisphenol FL; BPPH, bisphenol PH; BPSMAE, bisphenol S MAE; BPSMPE, bisphenol S MPE; BPTMC, bisphenol TMC; BTUM, bisphenol BTUM; BPSIP, 4-hydroxyphenyl 4-isopropoxyphenylsulfone; DD70, 1,7-bis(4-hydroxyphenylthio)-3,5-dioxaheptane; DD90, bis(2-chloroethyl)ether-4,4′-dihydroxydiphenyl sulfone copolymer; CTL = control; g [95% CI] = standardised mean difference with the Hedges’ g correction along with its 95% confidence interval; *I* ^2^ = Heterogeneity index; 95% PI = Prediction interval with its 95% confidence interval.

Platelet aggregation data from BPA, and the bisphenols with the greatest (BPF) and least (BPAF) pro-coagulant effects, were also compared with BPA’s new alternatives proposed as “safe”. Although the pooled effect depicted an effect favouring the CTL (SMD*g*: −0.15; _95%_CI: −0.98–0.68), the meta-analysis clearly showed two distinct aggregatory effects: 1) enhancement of ADP-induced platelet aggregation (SMD*g*: 1.93; _95%_CI: 1.35–2.50) by BPA, BPAF and BPF, and 2) a reduction in AA-induced platelet aggregation (SMD*g*: −0.73; _95%_CI: −1.41 to −0.04). Of note were the nuanced effects produced by bisphenols such as BP (SMD*g* = −0.26), C2 (SMD*g* = −0.37), SMPE (SMD*g* = 0.23), TMC (SMD*g* = 0.41), TUM (SMD*g* = −0.32), and DD90 (SMD*g* = 0.15) (Figure 5). Consistent with the prior meta-regression (R^2^ = 0.61), the two antagonists had opposite effects (ADP, β = 1.94, *p* ≤ 0.001; AA, β = −2.66, *p* ≤ 0.0001) (Supplementary Table S4), with residual heterogeneity (*I* ^*2*^ = 88.30%). The Baujat plot identified the effect of BPPH as a potential outlier, to a greater extent than BPA, BPAF, and BPF (data not shown). Nevertheless, were this bisphenol to be removed, heterogeneity would not show a notable difference, as it depended on the different EDCs tested. Regarding the effect of these new “safe” alternatives on PT at 100 μM, most of them prolonged the clotting time (SMD*g*: 0.36; _95%_CI: 0.02–0.71). However, BPSMPE exhibited an enhanced platelet aggregation SMD*g*: −0.97; _95%_CI: −1.57 to −0.37 (Supplementary Figure S4). These effects were greater at 300 μM (SMD*g*: 0.77; _95%_CI: 0.21–1.33), at which DD70 exhibited an enhanced platelet aggregation (SMD*g*: −1.51; _95%_CI: −3.33–0.30). The aPTT test at 100 μM showed a nuanced pooled effect on clotting time (SMD*g*: 0.04; _95%_CI: −1.07–1.15) with a broad _95%_CI that encompassed both prolonged and shortened clotting times (*i.e.*, BTUM, BPSIP, DD70, and DD90); similar patterns (SMD*g*: 0.41; _95%_CI: −0.70–1.52) were found at 300 μM (Supplementary Figure S4). The scanty results from *in vivo* studies also support the haemototoxic effects of bisphenols on PT, aPTT, bleeding time, blood clotting time and fibrinogen (Supplementary Table S1).

### 3.2 Differential bisphenols’ binding to key platelet receptors: results from *in silico* analyses

To explain our findings, *in silico* analyses of the most probable platelet receptors, based on their agonist chemical structures and their similarity to bisphenols, were performed.

#### 3.2.1 Validation of the 3D-PAR4 model and molecular docking

**FIGURE 6:**
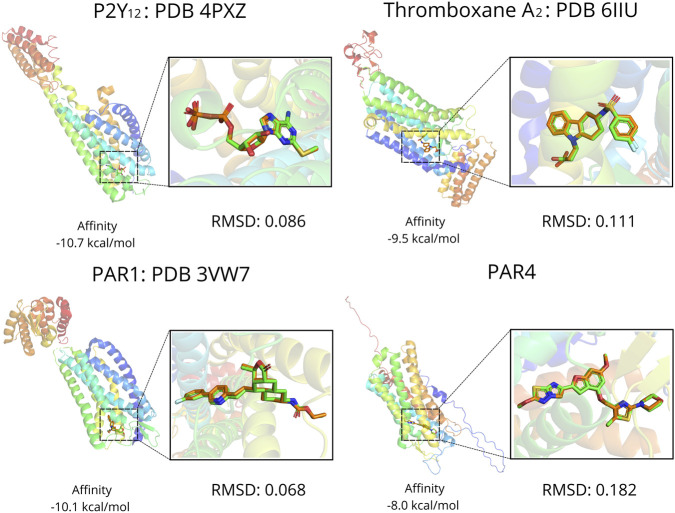
Molecular docking accuracy validation between the target receptors and their native ligands using Root-Mean-Square deviation analysis. Footnote: P2Y_12_, purinergic P2Y-subtype-12 receptor; PAR1, protease-activated 1 receptor; PAR4, protease-activated 4 receptor; RMSD, Root-Mean-Square deviation. The protein is depicted using rainbow colours with the N-terminus in blue and the C-terminus in red. A structural alignment between the native and re-docked ligands was performed to assess docking accuracy. Native ligands were displayed with the carbon colour code in green; the redocked ligands appear with the carbon atoms in orange.

The PAR4-3D-generated model showed high reliability (GMQE = 0.80, reference range: 0–1), good stereochemical quality (MolProbity score = 1.63), and a high proportion of residues correctly localised in favoured regions of the Ramachandran plot (90.08%). These quality metrics pertain to the complete PAR4 structural model obtained with AlphaFold-derived templates via Swiss-Model, rather than exclusively to the docking-relevant binding site region. Although some structural uncertainty may persist in flexible extracellular loops and in peripheral receptor regions, the orthosteric binding pocket retained an adequate local geometry. This geometry did not exhibit major steric clashes or unfavourable conformations that could compromise ligand docking analyses. These features, along with the other receptors and their respective agonists validation, exhibited RMSD values (≤2.0 Å) indicating adequate overlap with the experimental conformation. The structural alignment between the native and re-docked ligands demonstrated that the docking methodology was accurate and reliable, reproducing the original ligand positions of the target receptors (Figure 6). Similar results were obtained with endogenous ligands and their inhibitors using AutoDock Vina (Docking score), in which the search box was explicitly defined around the co-crystallised ligand-binding site based on coordinates, yielding favourable binding affinity values (Table 1). A comparable trend was observed in the binding affinities obtained with SwissDock (calculated affinity), even though the binding pocket was not manually defined. Of note that the binding affinity energies for the complex PAR1-thrombin (−11.70 kcal/mol) were more favourable than those obtained for the complex PAR4-thrombin (−9.40 kcal/mol).

**TABLE 1:** Binding affinity values of the endogenous ligands, the antagonists, and bisphenols on the target receptors.

​	Target receptors							
	P2Y_12_		TP		PAR1		PAR4	
Endogenous ligands	Antagonists, and bisphenols	Docking score	Calculated affinity	Docking score	Calculated affinity	Docking score	Calculated affinity	Docking score	Calculated affinity
		(kcal/mol)	(kcal/mol)	(kcal/mol)	(kcal/mol)	(kcal/mol)	(kcal/mol)	(kcal/mol)	(kcal/mol)
Endogenous ligands	ADP	−9.50	−8.70	-	-	-	-	-	-
	TxA_2_	-	-	−7.90	−7.10	-	-	-	-
	Thrombin	-	-	-	-	−11.70	−9.70	−9.40	−7.70
Antagonists	Cangrelor	−7.10	−6.70	-	-	-	-	-	-
	Ramatroban	-	-	−9.50	−9.30	-	-	-	-
	Vorapaxar	-	-	-	-	−10.10	−9.50	-	-
	Bms-986120	-	-	-	-	-	-	−7.80	−7.50
Bisphenols	BPA	−6.30	−5.95	**−8.10**	−7.04	−8.00	−7.02	−5.50	−6.25
	BPF	**−7.60**	**−6.57**	−7.20	−6.86	−7.70	−6.72	−6.40	−5.95
	BPS	**−7.40**	−6.31	**−7.50**	**−7.44**	−7.80	−7.00	−5.90	−6.35
	BPAF	−6.40	−5.95	**−8.20**	**−7.08**	−8.70	−7.33	−4.50	−6.17
	TDP	−6.50	**−6.69**	−6.90	**−7.18**	−7.10	−6.89	−5.80	−6.15

Footnote: In bold-green valour with binding affinities close to the endogenous ligands, whereas in bold-red valour with binding affinities close to the antagonists. BPA, Bisphenol A; BPF, Bisphenol F; BPS, Bisphenol S; BPAF, Bisphenol AF; TDP, 4,4′-thiodiphenol; P2Y_12_, purinergic P2Y-subtype-12, receptor; PAR1, protease-activated 1 receptor; PAR4, protease-activated 4 receptor, TP, thromboxane A_2_ receptor.

Regarding the binding affinity between the other receptors (P2Y_12_ and TP) and their specific agonists, Ramatobran showed the highest binding affinity (−9.50 kcal/mol) to unit TP, even higher than that of the endogenous ligand (−7.90 kcal/mol). Vorapaxar also showed a good score (−10.10 kcal/mol); however, it was not higher than that of the endogenous ligand for PAR1 (−11.70 kcal/mol).

#### 3.2.2 BPA’s structural analogues have more affinity for TP than its endogenous ligand

The docking analyses (Table 1) revealed that bisphenols could have more favourable binding affinity energies for TP than for its endogenous agonist. BPAF (−8.20 kcal/mol) and BPA (−8.10 kcal/mol) exhibited lower energy scores than TxA_2_ (−7.90 kcal/mol). Additionally, SwissParams values for BPS (−7.44 kcal/mol) and TDP (−7.18 kcal/mol) also had greater affinity for TP than their endogenous ligand (−7.10 kcal/mol); BPA and BPAF showed values like TxA2. The affinity of bisphenols for TP could explain the procoagulant effect observed in the *ex vivo* model.

Regarding P2RY_12_, BPF (−7.60 kcal/mol) and BPS (−7.40 kcal/mol) exhibited lower scores than Cangrelor (−7.10 kcal/mol), the reference antagonist. For Protease-Activated Receptors, bisphenols do not show preferent binding, since their scores range 1.35–1.65 and 1.47–2.09 times lower for PAR1 and PAR4, respectively, when compared to thrombin.

**FIGURE 7:**
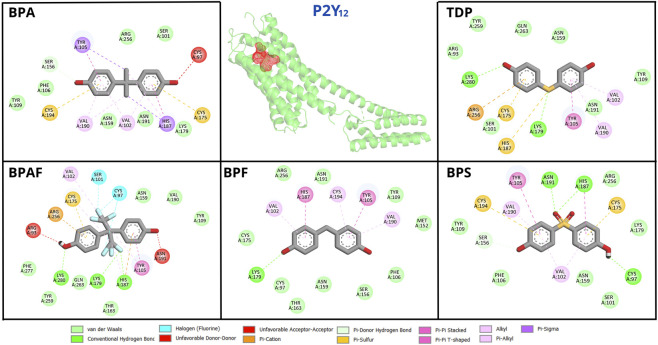
Predicted binding interaction of bisphenols and their parent compound with the binding pocket of the purinergic P2Y-subtype-12 receptor. Footnote: The three-dimensional structure of the purinergic P2Y-subtype-12 receptor is depicted in green, with the predicted binding pocket highlighted in red. BPA, bisphenol A; BPAF, bisphenol AF; BPF, bisphenol F; BPS, bisphenol S; TDP, 4,4′-thiodiphenol.

**FIGURE 8:**
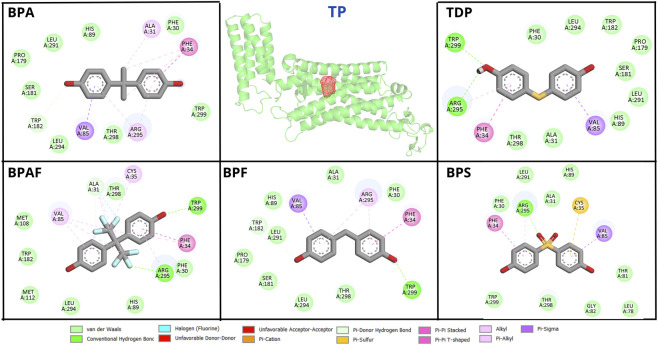
Predicted binding interaction of bisphenols and their parent compound with the binding pocket of the thromboxane A_2_ receptor. Footnote: The three-dimensional structure of the thromboxane A_2_ receptor is depicted in green, with the predicted binding pocket highlighted in red. BPA, bisphenol A; BPAF, bisphenol AF; BPF, bisphenol F; BPS, bisphenol S; TDP, 4,4′-thiodiphenol.

The amino acid residues involved in the molecular recognition of both endogenous ligands and antagonists showed a remarkable conservation within the binding sites for TP (70%) and P2Y_12_ (64%) (Supplementary Table S5). This conservation was found mainly in TP (70%) and P2RY_12_ (64%); PAR1 and PAR4 exhibited discrepant recognition patterns. However, the evaluation of amino acid residues involved in binding interactions between the bisphenols and the target receptors revealed that several residues shared both agonist and antagonist interactions (Supplementary Tables S6-S9; Supplementary Figure S5). Although BPF showed the highest binding affinity for P2RY_12_, BPAF had the greatest number of molecular interactions with active-site residues (Figure 7). As mentioned before, BPAF showed the highest affinity for TP; however, all bisphenols form the same number of interactions −13 — with this receptor (Figure 8).

**FIGURE 9:**
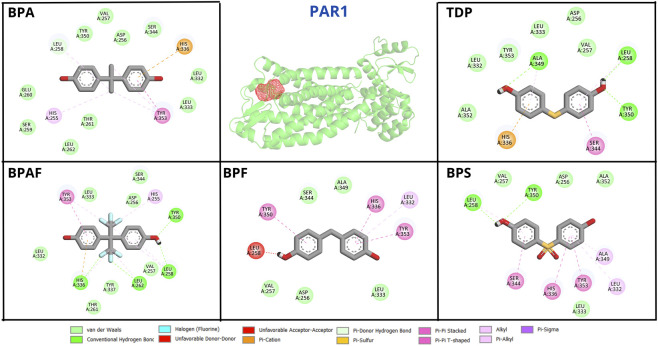
Predicted binding interaction of bisphenols and their parent compound with the binding pocket of the protease-activated 1 receptor. Footnote: The three-dimensional structure of the protease-activated 1 receptor is depicted in green, with the predicted binding pocket highlighted in red. BPA, bisphenol A; BPAF, bisphenol AF; BPF, bisphenol F; BPS, bisphenol S; TDP, 4,4′-thiodiphenol.

**FIGURE 10:**
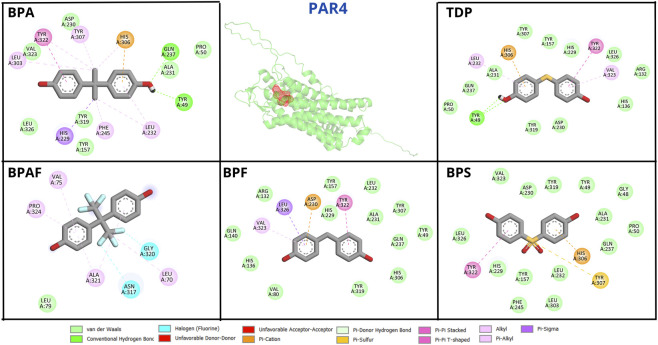
Predicted binding interaction of bisphenols and their parent compound with the binding pocket of the protease-activated 4 receptor. Footnote: The three-dimensional structure of the protease-activated 4 receptor is depicted in green, with the predicted binding pocket highlighted in red. BPA, bisphenol A; BPAF, bisphenol AF; BPF, bisphenol F; BPS, bisphenol S; TDP, 4,4′-thiodiphenol.

Bisphenols showed differential affinity for PAR1 (BPA and BPAF shared 70% of interactions with the antagonist); in contrast, for PAR4, bisphenols shared around 50% of interactions with the antagonist site (Figures 9, 10). Interestingly, whereas BPF shaped almost 60% interactions with the ligand-binding site of PAR4, BPAF did not interact with the antagonist Bms-986120 binding site (Supplementary Table S9).

## 4 Discussion

Cardiovascular burden will remain an urgent global health concern that must be addressed through multiple guidelines. Atherosclerosis, endothelial dysfunction, hypertension, and increased carotid intima-media thickness have been documented as effects on cardiovascular health from environmental xenobiotic exposure (Palacios-Valladares et al., 2024; Munzel et al., 2025). Nonetheless, studies on the impact of xenobiotics on thrombosis are scarce. The underlying cause of MI, stroke, critical limb ischaemia, and venous thromboembolism is thrombosis. In the present study, the effects of BPA and its most used structural analogues (BPF, BPS, BPAF), as well as its parent compound—TDP—, on platelet aggregation were assessed using an *ex vivo* model. *In silico* analyses were performed to explain and identify potential receptors through which bisphenols induce a significant increase in platelet aggregation. Ultimately, our findings were compared with several studies from a systematic review spanning 10 years.

### 4.1 Bisphenols and their contribution to platelet aggregation

Although 1% DMSO did not affect ADP-induced platelet aggregation in our experimental system, DMSO has been reported to modulate platelet function, depending on the agonist, concentration, incubation time, and platelet preparation (Mendes-Silverio et al., 2012; Bolibrukh et al., 2015). In our study (1%) and in the second-largest study (0.80%), the DMSO concentrations used were not notably different (Hrubsa et al., 2025; Hrubsa et al., 2026). Nonetheless, some discrepancies were observed. Meta-regressions revealed divergent effects of BPA on ADP-induced platelet aggregation (increase) versus AA-induced platelet aggregation (decrease), which might explain the remarkable heterogeneity introduced into the meta-analysis by Hrubsa’s study (Hrubsa et al., 2026). A discrepant effect was also observed regarding platelet aggregate stability: our study demonstrated sustained platelet aggregation, whereas another study using BPAF did not (Vishalakshi et al., 2021). These differences could be explained by the biphasic effect reported for some bisphenols (BPAF), which is concentration-dependent (Vishalakshi et al., 2021). Of note was also the increase in platelet aggregation alongside prolonged clotting times. This apparent paradox suggests that bisphenols, as well as other EDCs, could act both in primary (platelets) and in secondary haemostasis (coagulation cascade) (Chagas et al., 2021; Vishalakshi et al., 2021; Fonseca et al., 2022). Although receptor interactions may contribute to platelet response, the lack of a simple monotonic profile suggests that additional mechanisms (*e.g*., oxidative stress, membrane effects, modulation of intracellular signalling, and release of secondary mediators) may also contribute (De Toni et al., 2020; Burgos et al., 2025). Analogous studies (*ex vivo* human platelets exposure) revealed an increased risk of blood clot formation by BPA and BPS (Chagas et al., 2021; Burgos et al., 2025). An increase in coagulation factors due to BPA effects, as demonstrated *in vivo* models, has also been proposed alongside decreased bleeding time, PT, and aPTT (Akhter et al., 2023). The concentration-response experiments indicated that the pro-aggregatory effect of bisphenols on ADP-induced platelet aggregation is compound-specific and may not follow a consistent pattern (Vandenberg, 2014). In addition, the present study tested low concentrations (range: pM to nM), whereas other studies tested μM concentrations. Such differences limited comparability across studies, resulting in high heterogeneity. Therefore, the interpretation of the vehicle effects should be restricted to the present experimental conditions.

Conversely, the blocking of certain clotting factors (*e.g*., Factor VIIa by BPA), a reduction in fibrinogen leading to prolonged PT, and interference with the intrinsic clotting pathway favoured the anticoagulant effect (Chagas et al., 2021). These results were reinforced by coagulation parameters such as PT and aPTT, even in the considered “safe” alternatives (Hrubsa et al., 2025; Hrubsa et al., 2026). Previous studies, summarised in the meta-analyses herein, reinforced the effects of these EDCs on platelet aggregation (pro- and anti-thrombotic) through *ex vivo* exposure. This effect has also been observed in the zebrafish exposed to high BPA concentrations (Chagas et al., 2021).

Furthermore, sex and age have been shown to influence platelet reactivity (Recabarren-Leiva et al., 2021; Gasecka et al., 2023). Consequently, our study design involved only men and platelet pools from several individuals, reducing certain biological variability. In our meta-analyses, sex was not a source of heterogeneity (*p* = 0.70), possibly because our study is the largest conducted to date, thereby influencing the weight of the pooled results. Other studies included more males than females (1.7:1 and 1.5:1), thereby reducing the influence of sex on the results (Hrubsa et al., 2025; Hrubsa et al., 2026). Variations in agonists, platelet preparation, exposure conditions, bisphenols’ concentrations, vehicles, and individual variability should also be considered. Thus, the findings from meta-analyses should be interpreted in light of these limitations. Moreover, our results did not generalise to women, which represents a relevant limitation of our study. Hence, further studies including female donors and non-healthy individual should be conducted. Furthermore, the effect of menstrual cycle phase, menopausal status, and hormone use, as well as the additional influence of bisphenols, should be considered in future studies. However, accordingly, the current body of evidence best supports the interpretation that bisphenols’ effects are context-dependent but consistently non-neutral with respect to haemostasis (Vishalakshi et al., 2021; Burgos et al., 2025).

In the context of CVDs, the bisphenols’ effects on platelet aggregation were clear, suggesting that these EDCs could contribute to their development. In the last 10 years, only eight studies, including this one, have reported the effects of bisphenols on thrombosis (Chagas et al., 2021; Vishalakshi et al., 2021; Akhter et al., 2023; Li et al., 2023; Burgos et al., 2025; Hrubsa et al., 2025; Hrubsa et al., 2026). Of these, 50% through BPA, followed by BPS (25%) and BPAF (12.50%), leaving aside BPF, the second-most-found bisphenol in urine (Duan et al., 2026). Our findings suggested that even at low concentrations (5 pM), BPF increased platelet aggregation, followed by BPA > BPS > and BPAF. This pro-aggregatory effect suggests that BPF may retain, or even exceed, the platelet-sensitising activity of BPA. This interpretation aligns with the broader toxicological literature, indicating that BPA and its analogues cannot be presumed biologically neutral. Although BPA has not shown AA-induced platelet aggregation, it has influenced PT (increased) and aPTT (decreased) coagulation parameters (Hrubsa et al., 2026). Therefore, the concept of “safe” should be used cautiously until comparative toxicodynamic and exposure data are available. The estimated tolerable daily intake (TDI) from the European Food Safety Authority (EFSA) for BPA is 0.20 ng/kg body weight/day (Vom Saal et al., 2024). Human studies have shown that the concentrations of BPA substitutes in urine samples (BPA > BPF > BPAF > BPS) far exceed established thresholds (Duan et al., 2026). In cardiovascular health, this is a critical point; both MI and stroke have shown an upward trend in young and middle-aged adults (Antza et al., 2023).

### 4.2 Possible mechanisms involved in the pro-aggregating effect of bisphenols

Our findings indicate that bisphenols can act as amplifiers of agonist-dependent platelet activation, leading to an exaggerated secondary wave. This pattern is compatible with a platelet-priming effect, where platelets remain apparently quiescent at baseline. Nonetheless, once stimulated, platelets respond disproportionately. Given that the P2Y_12_ axis is a cornerstone in the amplification and stabilisation of platelet aggregation, the fact that bisphenols enhanced the secondary wave is particularly informative. The second phase of ADP-induced aggregation depends on dense-granule secretion, release of endogenous ADP, sustained P2Y_12_-dependent signalling, TxA_2_ generation, calcium mobilisation, and stabilisation of αIIbβ3-mediated platelet-platelet interactions (Gachet, 2012). Hence, the tracing profile observed after bisphenol exposure suggests that these compounds may act on platelet amplification loops, leading to platelets appearing more reactive and less readily reversible once stimulated (Gachet, 2012). A compound that accelerates dense-granule secretion or thromboxane generation would not necessarily induce spontaneous aggregation; yet it would be expected to amplify the secondary wave after ADP stimulation. This functional phenotype is consistent with enhanced thrombogenic potential, and in the context of arterial thrombosis, such a shift may be highly relevant, since thrombus stability depends on these reinforcing pathways (Gachet, 2012; Palacios-Valladares et al., 2024).

Given that bisphenols are hydrophobic phenolic molecules, they could also alter the platelet membrane environment, partition into the lipid bilayer, and modify membrane fluidity, receptor mobility, or the organisation of signalling microdomains (Rashid et al., 2017; Li et al., 2023). In this setting, bisphenols would not need to act as classical receptor agonists and could facilitate signal propagation by altering the membrane environment in which platelet receptors operate (Palacios-Valladares et al., 2024). Reactive oxygen species (ROS) and ROS-dependent signalling are associated with platelet activation, granule secretion, and pro-coagulant activity, which, *per se*, could also explain our findings. BPA, BPS, and BPAF induce oxidative stress in platelets, increasing ROS in both cytosol and mitochondria (Vishalakshi et al., 2021; Li et al., 2023; Burgos et al., 2025). Platelets are highly sensitive to redox changes, and moderate increases in oxidative tone can enhance calcium signalling, secretion, phospholipase activity, thromboxane production, integrin activation, and phosphatidylserine exposure. A recent platelet-focused study reported that BPA enhances platelet activation and aggregation via oxidative stress and kinase-associated pathways, reinforcing the biological plausibility of this mechanism (Burgos et al., 2025). In addition, modifications in platelet-derived extracellular vesicle cargo, as a result of bisphenol exposure, alter the transcriptomic profile of genes related to platelet function and thrombogenicity (Ali et al., 2025; Kubaev et al., 2025; Pedersen et al., 2025).

Another attractive and plausible mechanism to explain the effects of bisphenols on platelet aggregation may involve binding to the active sites of platelet receptors, a hypothesis explored herein through *in silico* analyses. Although platelets are anucleate, they retain multiple membrane receptors and signalling networks that enable them to respond quickly to extracellular stimuli; bisphenols could modulate non-genomic steroid-like signalling pathways.

#### 4.2.1 Possible mechanisms through TP receptor

Our study is the first to demonstrate — *in silico* — the favourable binding affinities of bisphenols for TP, comparable to or even higher than those of its endogenous ligand. BPAF was the structural analogue with the highest affinity binding (−8.20 kcal/mol) for TP regarding TxA_2_ (−7.90 kcal/mol). Nonetheless, its effect on platelet aggregation was nuanced. Exposure to low concentrations of BPAF has been shown to increase P-selectin, an adhesion molecule with a critical role in thrombosis and inflammation (Vishalakshi et al., 2021). Platelet shape modification is a signature of receptor activation (*e*.*g*., TxA_2_/TP). Cytoskeletal rearrangements in platelets, such as protrusions that promote platelet aggregation, may require higher concentrations, as demonstrated by a prior study exposing *ex vivo* human platelets to higher concentrations (25 µM) of BPAF (Vishalakshi et al., 2021).

Our findings were also supported by a previous study, which reported a remarkable affinity of bisphenol A bis (diphenyl phosphate, BDP) for TP (−9.00 kcal/mol). In addition, BDP also increased (dose-dependent) platelet adhesion and aggregation, as well as P-selectin fluorescence intensity (Chen et al., 2025). In such circumstances, it is likely that bisphenols maintain platelet activation, similar to that caused by TxA_2_ binding its receptor. Persistent TxA_2_-dependent-platelet activation has been associated with accelerated atherogenesis, whereas increased TxA_2_ production was related to cardiac fibrosis and platelet extravasation in the heart (Patrono et al., 2026). Hyperreactive platelet activation has been associated with occlusive thrombus pathologies such as acute coronary syndrome, MI and stroke (Amison and Pitchford, 2026). Although BPAF showed the highest binding affinity, other bisphenols, such as BPA, BPS, and BPF, could compete for the binding site at higher concentrations.

In prior studies, BPA and two structural analogues (DD-70 and BPH), have shown an inhibitory effect on cyclooxygenase 1 (COX-1) (Hrubsa et al., 2025; Hrubsa et al., 2026). This effect could suppress TxA_2_-dependent platelet activation. Yet, aggregation and coagulation are complex processes in which several receptors, in addition to COX-1, mediate the production of TxA_2_ and other prostanoids (Patrono et al., 2026). For this reason, antiplatelet agent combinations are clinically used to “control” the hyperactive platelet activation (Amison and Pitchford, 2026). Xenobiotic doses also play a critical role; low-dose BPA has been shown to upregulate COX-2 and prostaglandin D_2_ (PGD_2_) synthase in Sprague-Dawley rats (Wu et al., 2016; Wu et al., 2020). These two possibilities could favour a pro-aggregating state. The interaction of bisphenols with other coagulation factors (i.e., FVIIa) through the extrinsic pathway supports the promiscuous effect of bisphenols in blood coagulation (Chagas et al., 2021).

Although TP could explain the *ex vivo* findings, the binding affinity did not align with platelet aggregation differences. Thus, several mechanisms could be involved, and each bisphenol may exhibit differential effects, possibly associated with its particular chemical structure. This is particularly challenging in the regulation of “BPA-free” products, which contain bisphenol mixtures that exceed established thresholds (Duan et al., 2026). Further studies are needed to understand the combined effects of these xenobiotics, using designs that better mimic real-world environmental exposure and elucidate their effects on cardiovascular health.

#### 4.2.2 Dual face of bisphenols on P2Y_12_ receptor

P2Y_12_ is one of the most therapeutically relevant targets for antithrombotic agents; thus, the binding affinity exhibited by bisphenols could have diverse pharmacological consequences. BPF (−7.60 kcal/mol) and BPS (−7.40 kcal/mol) exhibited higher binding affinity energies for P2Y_12_ than the potent P2Y_12_ inhibitor cangrelor (−7.10 kcal/mol). Cangrelor is the most effective antiplatelet drug compared to clopidogrel, prasugrel, and ticagrelor, with irreversible action. It has an immediate effect (intravenous administration) on platelet aggregation, which reverses to normal platelet function within one or 2 h (Hendrianus et al., 2025).

P2Y_12_ antagonists inhibit ADP activity and, in turn, platelet aggregation via G_αi2_ protein. Also, P2Y_12_ antagonist inhibits Ras GTPase-activating protein 3 (RASA3), thereby promoting Rap1b GTPase activity and integrin activation (von Kugelgen, 2017). Nucleoside analogues, such as thienopyridines and ATP, act as P2Y_12_ antagonists. Thus, it is not surprising that bisphenols, sharing some structural analogy, could have a similar function. The P2Y_12_ inactive binding pocket is a cavity within the receptor to which antagonists can bind. It has two subpockets; AZD1283, a high-potency antiplatelet aggregation drug, binds to subpocket-1 (transmembrane, TM, regions 3–7) via the amino acid residues Arg256 and Lys280. Clopidogrel and prasugrel interact with the residue Cys97 (TM-3), forming a disulfide bridge with Cys175 (extracellular loop-2) (von Kugelgen, 2017). All bisphenols exhibited a high probability to bind to Arg256, whereas BPAF and TDP bind to Lys280. The disulfide bridge is highly conserved; all bisphenols bind to Cys97 and Cys175, which could confer an antiplatelet-drug-like effect. As a G-protein-coupled receptor, P2Y_12_ undergoes large-scale rearrangements; the agonist also binds subpocket-1, where its negative charge interacts with positively charged amino acid residues (Ullah et al., 2025). Arg93 (BPAF and TDP), Arg256 (all bisphenols tested herein), Lys280 (BPAF and TDP), and Tyr105 (all bisphenols tested herein) are shared between agonist and bisphenols, suggesting also a pro-coagulant effect (von Kugelgen, 2017). BDP, an organophosphate ester, showed close affinity by P2Y_12_ (−8.23 kcal/mol), reinforcing the possibility that bisphenols could also act as agonists (Chen et al., 2025).

Thus, bisphenols could play a pivotal role in haemostasis; as antagonists, these EDCs may increase the risk of bleeding. Pharmacologically, this may have adverse consequences, a critical point even in the thrombotic therapy; cangrelor has been associated with 1.5 times more bleeding events than clopidogrel (Prasad et al., 2025). As a possible mechanism, bisphenols could enhance endogenous fibrinolysis, as cangrelor does, predisposing, *per se*, to bleeding events (Prasad et al., 2025). Bisphenols could also act as competitive P2Y_12_ receptor antagonists, altering the effects of antiplatelet drugs. Cangrelor, tricagrelor, diadenosine tetraphosphate derivatives and uridine triphosphate thio-analogues are potent and competitive P2Y_12_ antagonists even at nanomolar concentrations (von Kugelgen, 2017). As agonists, they could also have a pro-coagulant effect. The antagonist ticagrelor has also shown an agonist effect, increasing extracellular adenosine concentrations by blocking nucleoside transporter-1 (von Kugelgen, 2017). Another possible mechanism is the P2Y_12_ relocalisation to the cell membrane, followed by desensitisation and internalisation in platelets; a mechanism described for ADP-P2Y_12_ binding (von Kugelgen, 2017). P2Y_12_ has also been associated with plaque formation underlying endothelial dysfunction, a well-established role of bisphenols’ exposure (Palacios-Valladares et al., 2024; Dabravolski et al., 2025). Thrombus formation is a consequence of atherosclerotic plaques, a pathological process known as atherothrombosis.

#### 4.2.3 Possible effects of bisphenols through PAR1 and PAR4

The more favourable binding affinity energies for PAR1-thrombin than for PAR4-thrombin was consistent with a prior report (Nieman and Schmaier, 2007). However, our *in silico* findings regarding PAR1 and PAR4 did not, *per se*, support platelet aggregation induced by bisphenols, as these compounds showed only partial occupation of PAR1’s orthosteric site. This interaction did not involve the key amino acids required to form the “tethered ligand”, necessary for triggering intracellular signalling (Lee-Rivera et al., 2022). Nonetheless, all bisphenols could sterically hinder the conformational rearrangements of Tyr350 and Tyr353. BPA and BPAF could also form hydrogen bonds between Tyr350 and His255, stabilising Tyr350 and partially inhibiting PAR1 activation, as observed with vorapaxar, a competitive antagonist of the “tethered ligand” that binds to TM regions 3–7 (Lyu et al., 2025). This partial inhibition could have pharmacological consequences, including haemorrhagic events, decreased haemoglobin, and vascular disorders akin to those described for vorapaxar (Zhang et al., 2026). Regarding PAR4, bisphenols exhibited low affinity, binding to amino acid residues involved in both agonist and antagonist binding sites. Nonetheless, platelet aggregation is mediated by several signalling pathways. Hence, this did not rule out a possible effect of bisphenols via PARs, which could transactivate other receptors, given their coupling to G_αq_, G_α12,13_ (like TP), G_αi_ (like P2Y_12_), and G_βγ_ (Fernandez et al., 2021; Lee-Rivera et al., 2022). Howbeit, these are speculative mechanisms that should be interpreted in light of these limitations.

Bisphenols could also act as allosteric modulators affecting the sensitivity of PAR1 and PAR4 to their orthosteric ligand, skewing signalling (Kendrick et al., 2025). Nonetheless, further experiments using multiple concentrations of receptor-specific antagonist should be conducted, which was a limitation of the present study. In addition, molecular dynamics simulations for these and other receptors represent a key complementary strategy for investigate the stability and dynamic behaviour of the protein-ligand complexes generated by docking.

## 5 Conclusion

Our findings, together with those of other researchers, highlighted bisphenols as highly toxic xenobiotics that act as haemostatic disruptors, sensitising human platelets to agonist stimulation. Certainly, our study raises more questions than it answers, yet it has identified a critical underserved gap. For the first time, our study illuminates the interactions of BPA and its most common structural analogues with key receptors involved in platelet aggregation. Nonetheless, the coagulation-thrombosis process is complex, depending not only on environmental exposure, but also on a range of host factors. Comorbidities such as age, sex, pre-existing metabolic conditions, lifestyle, family history, and individual variability both in platelet reactivity and metabolic capacity (*e.g*., CYP2C19) play a critical role. It is important to emphasise that because “BPA-free” products contain bisphenol mixtures, further studies representing real-world human exposure, rather than the effects of individual bisphenols should be conducted.

Although *in silico* approaches provide valuable approximations of a molecule’s global properties, further studies on the effects of bisphenols should be conducted across different models to elucidate the mechanisms of action. Given that the mechanisms underlying these receptors may depend on ligand concentration, more studies using antagonists should also be conducted.

The current evidence pointed out that none of the present-day BPA substitutes is safe, emphasising the necessity to regulate their indiscriminate use in susceptible populations such as newborns, children and adolescents.

## Data Availability

The original contributions presented in the study are included in the article/Supplementary Material, further inquiries can be directed to the corresponding author.

## Supplementary material

SUPPLEMENTARY FIGURE S1 Effect of several concentrations of dimethyl sulfoxide on platelet aggregation induced with four agonists. CTL = control; C1 = 0.0001%; C2 = 0.001%; C3 = 0.01%; C4 = 0.1%; C5 = 1%, ADP [5μM], collagen [2μg/mL], epinephrine [1.1μM], and 1U of thrombin. Data are presented as mean ± standard error of the mean (SEM). The statistical differences among the concentrations tested were analysed with one-way ANOVA with Dunnett’s *post hoc* test relative to the CTL group.

SUPPLEMENTARY FIGURE S2 Effect of bisphenols (without agonist) on platelet aggregation. CTL (+) = ADP [5μM]; collagen [2μg/mL]; epinephrine [1.1μM]; and 1U of thrombin, CTL (-) = Platelet-rich plasma without agonist; BPA = bisphenol A; BPAF = bisphenol AF; BPF = bisphenol F; BPS = bisphenol S; TDP = thiodiphenol. Data are presented as mean ± standard error of the mean (SEM). The statistical differences among the concentrations tested were analysed with one-way ANOVA with Dunnett’s *post hoc* test relative to the CTL group.

SUPPLEMENTARY FIGURE S3 Pooled and group meta-analyses of the effect of bisphenol A on platelet aggregation along, with influence analysis. **(A)** Forest plot of the pooled effect of bisphenol A. **(B)** Forest plot by agonist groups of the effect of bisphenol A. **(C)** Influence analyses represented with the Baujat plot. CTL = control; g [95% CI] = standardised mean difference with the Hedges’ g correction along with its 95% confidence interval; *I* ^2^ = Heterogeneity index; 95% PI = Prediction interval with its 95% confidence interval.

SUPPLEMENTARY FIGURE S4 Forest plots of the effect of new alternative bisphenols on prothrombin time and activated partial thromboplastin time at two exposure concentrations. **(A)** Forest plot of the pooled effect of the 100μM concentration of new alternative bisphenols on prothrombin time. **(B)** Forest plot of the pooled effect of the 100μM concentration of new alternative bisphenols on activated partial thromboplastin time. **(C)** Forest plot of the pooled effect of the 300μM concentration of new alternative bisphenols on prothrombin time. **(D)** Forest plot of the pooled effect of the 300μM concentration of new alternative bisphenols on activated partial thromboplastin time. CTL = control; g [95% CI] = standardised mean difference with the Hedges’ g correction along with its 95% confidence interval; *I* ^2^ = Heterogeneity index; 95% PI = Prediction interval with its 95% confidence interval.

SUPPLEMENTARY FIGURE S5 Amino acid residues in both agonist and antagonist binding sites involved in binding interactions of target receptors. P2Y_12_, purinergic P2Y-subtype-12 receptor; TP, thromboxane A_2_ receptor; PAR1, protease-activated 1 receptor; PAR4, protease-activated 4 receptor.

SUPPLEMENTARY TABLE S1 Dimensions and centre coordinates of the docking boxes’ for each receptor included in the present study. P2Y_12_, purinergic P2Y-subtype-12 receptor; TP, thromboxane A_2_ receptor; PAR1, protease-activated 1 receptor; PAR4, protease-activated 4 receptor.

SUPPLEMENTARY TABLE S2 Summary of the results from papers included in the present meta-analyses. Numbers in red were obtained from plots and represent approximations. In the *in vivo* study by Chagas-Paes *et al.*, the N is an average and is also shown in red.

SUPPLEMENTARY TABLE S3 Parameters obtained from the meta-regression of bisphenol A effects on platelet aggregation with respect to agonists and leave-one-out results.

SUPPLEMENTARY TABLE S4 Parameters obtained from the meta-regression of bisphenols A, AF, and F compared with the new alternative bisphenols’ effects on platelet aggregation, with respect to agonists and leave-one-out results.

SUPPLEMENTARY TABLE S5 Amino acid residues involved in the molecular recognition of both endogenous ligands and antagonists. AA, amino acid; P2Y_12_, purinergic P2Y-subtype-12 receptor; TP, thromboxane A_2_ receptor; PAR1, protease-activated 1 receptor; PAR4, protease-activated 4 receptor.

SUPPLEMENTARY TABLE S6 Amino acid residues involved in binding interactions between the bisphenols and the purinergic P2Y-subtype-12 receptor. BPA, bisphenol A; BPF, bisphenol F; BPS, bisphenol S; BPAF, bisphenol AF; TDP, 4,4’-thiodiphenol, parent compound.

SUPPLEMENTARY TABLE S7 Amino acid residues involved in binding interactions between the bisphenols and the thromboxane A2 receptor. BPA, bisphenol A; BPF, bisphenol F; BPS, bisphenol S; BPAF, bisphenol AF; TDP, 4,4’-thiodiphenol, parent compound.

SUPPLEMENTARY TABLE S8 Amino acid residues involved in binding interactions between the bisphenols and the protease-activated receptor 1. BPA, bisphenol A; BPF, bisphenol F; BPS, bisphenol S; BPAF, bisphenol AF; TDP, 4,4’-thiodiphenol, parent compound.

SUPPLEMENTARY TABLE S9 Amino acid residues involved in binding interactions between the bisphenols and the protease-activated receptor 4. BPA, bisphenol A; BPF, bisphenol F; BPS, bisphenol S; BPAF, bisphenol AF; TDP, 4,4’-thiodiphenol, parent compound.
