# Supplementary figures and images for: Bisphenols-enhanced platelet aggregation via TP, P2Y12, PAR1 and PAR4 receptors: a thrombotic legacy in a plastic-ubiquitous world
Source: Front Pharmacol. 2026 Jun 22;17:1840963. doi: 10.3389/fphar.2026.1840963 (PMC13333404; doi:10.3389/fphar.2026.1840963)

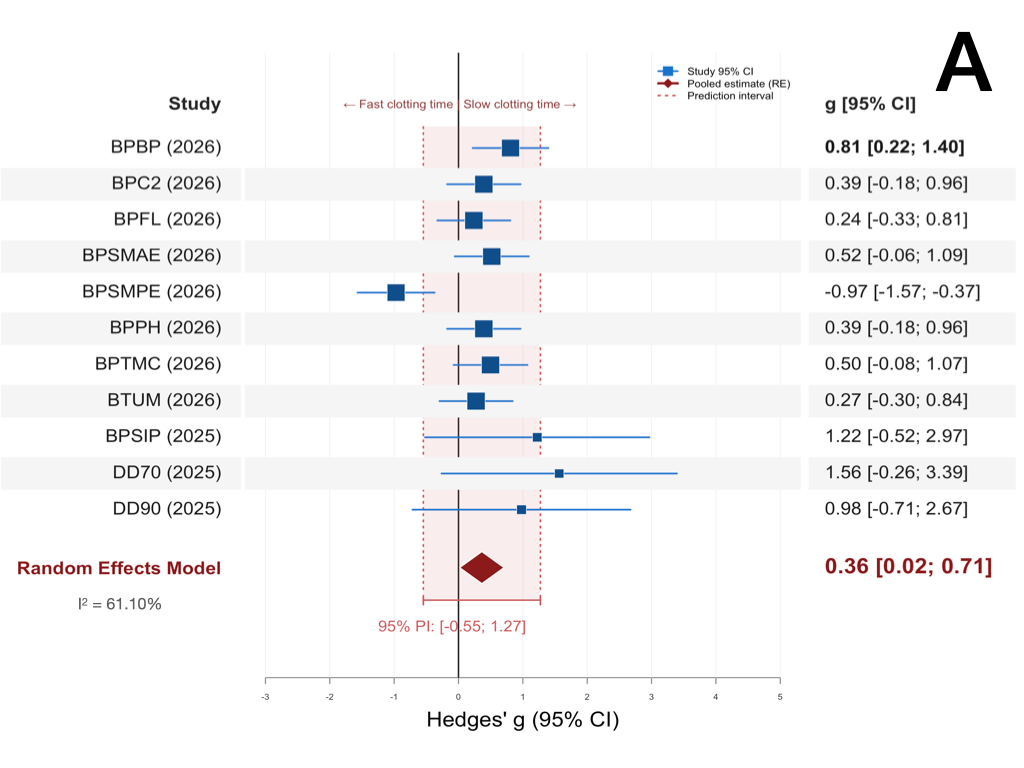

Supplement: Supplementary file 1 [file Image6.tif]

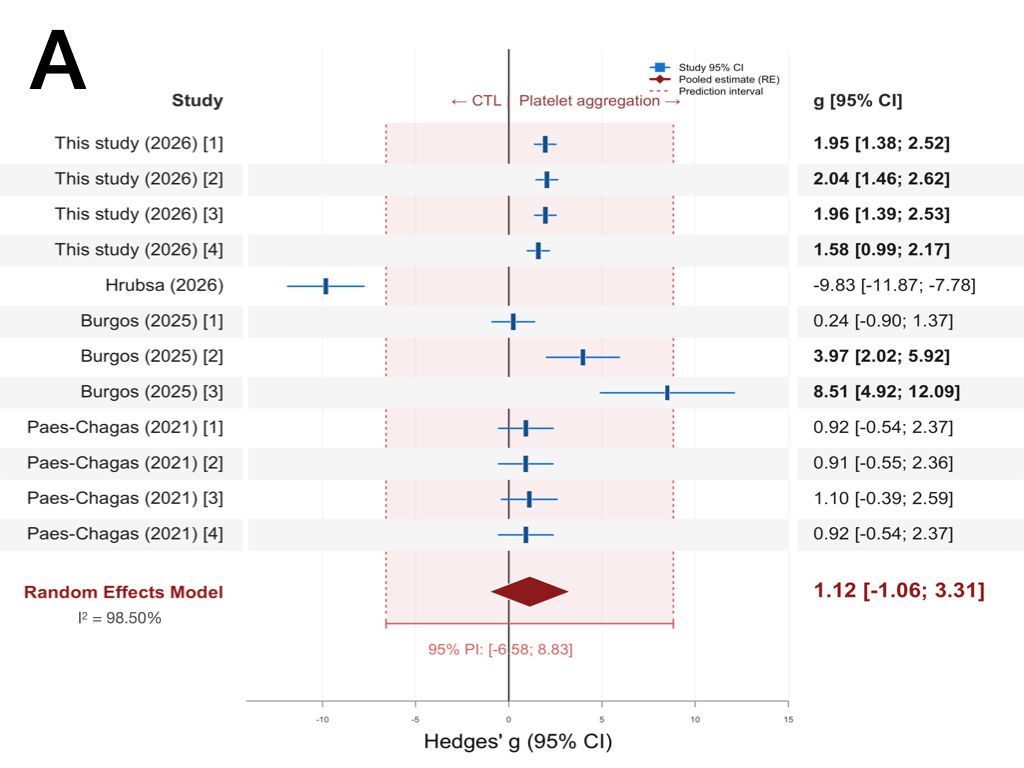

Supplement: Supplementary file 2 [file Image3.tif]

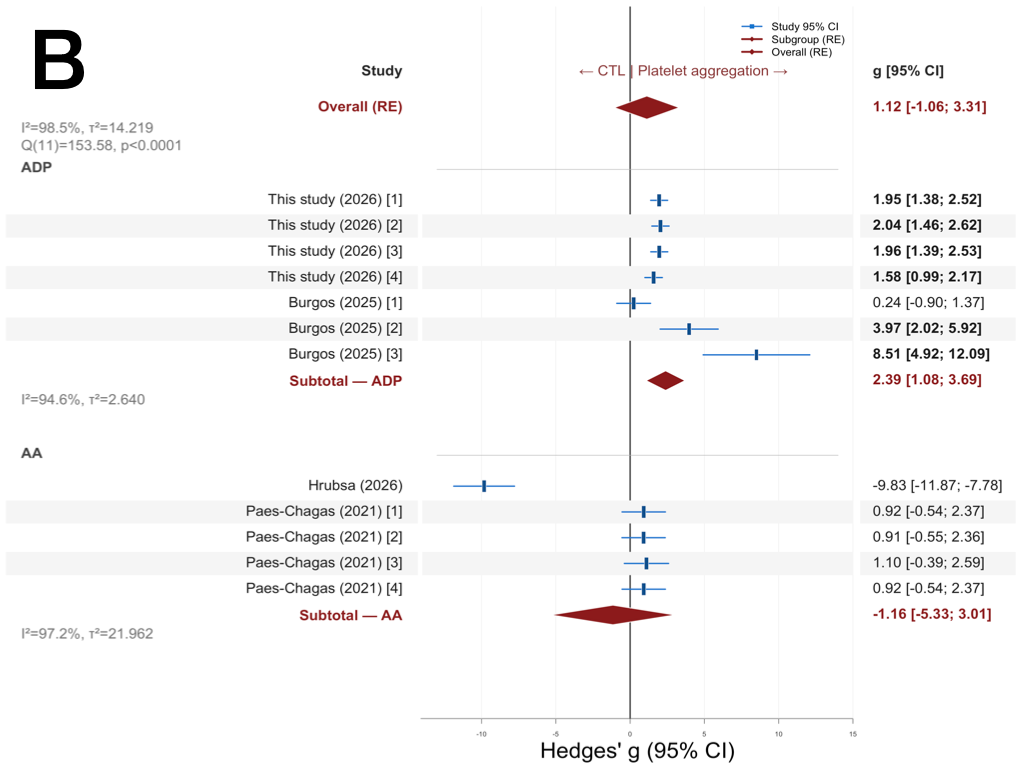

Supplement: Supplementary file 3 [file Image4.tif]

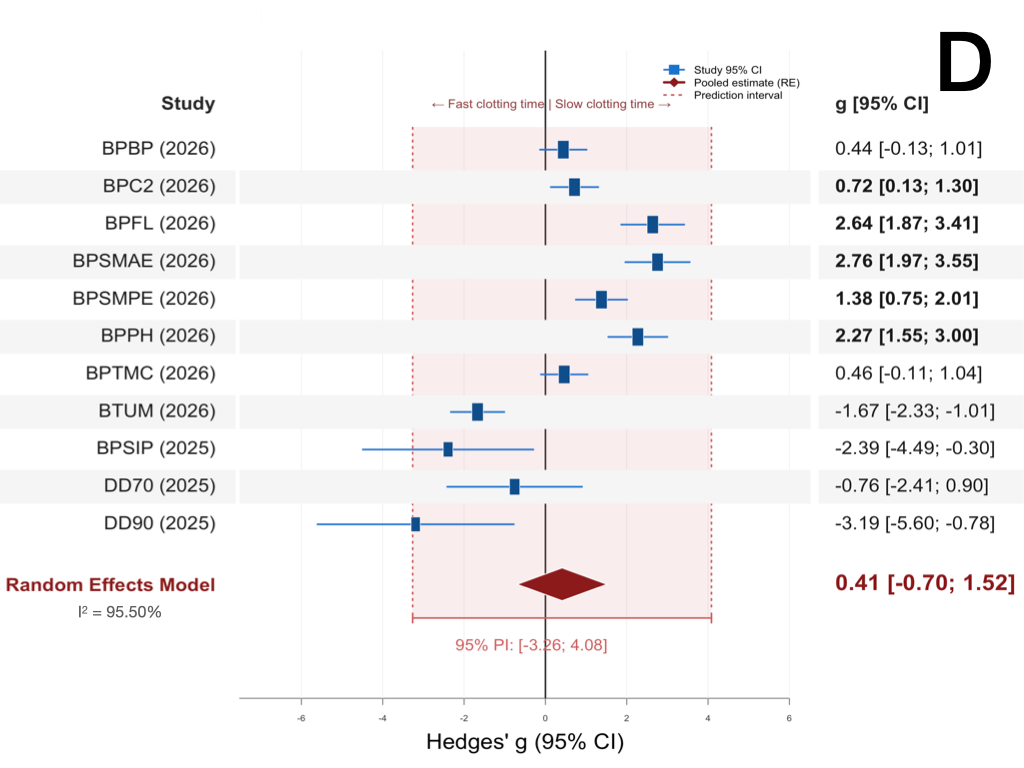

Supplement: Supplementary file 4 [file Image9.tif]

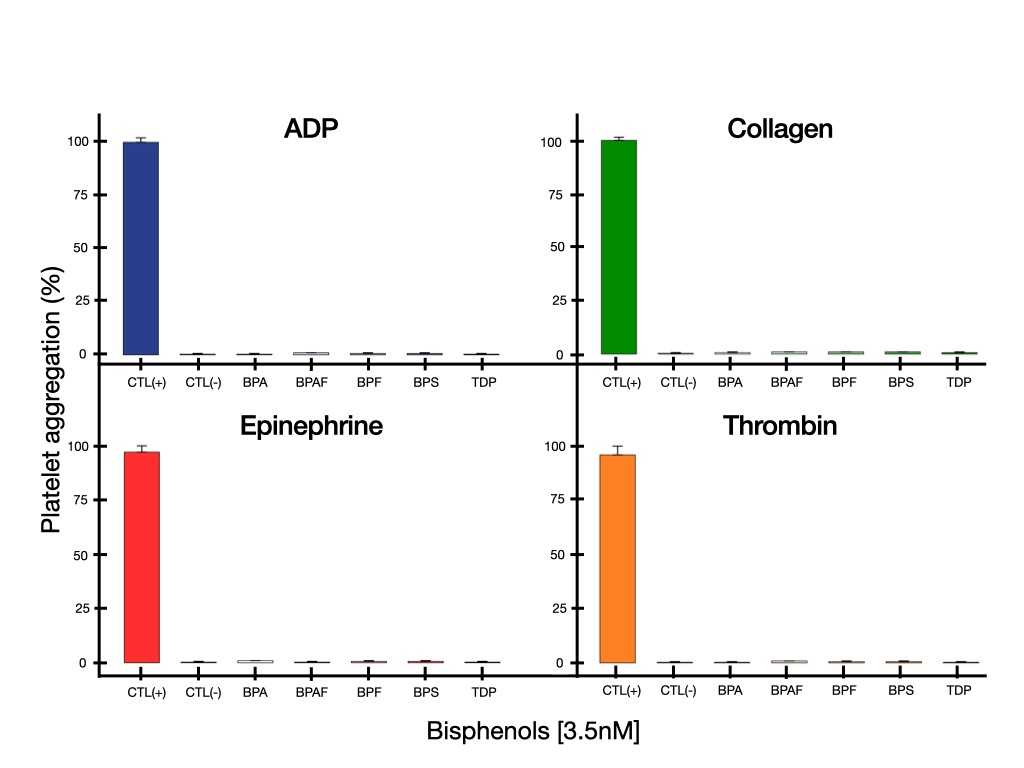

Supplement: Supplementary file 5 [file Image2.tif]

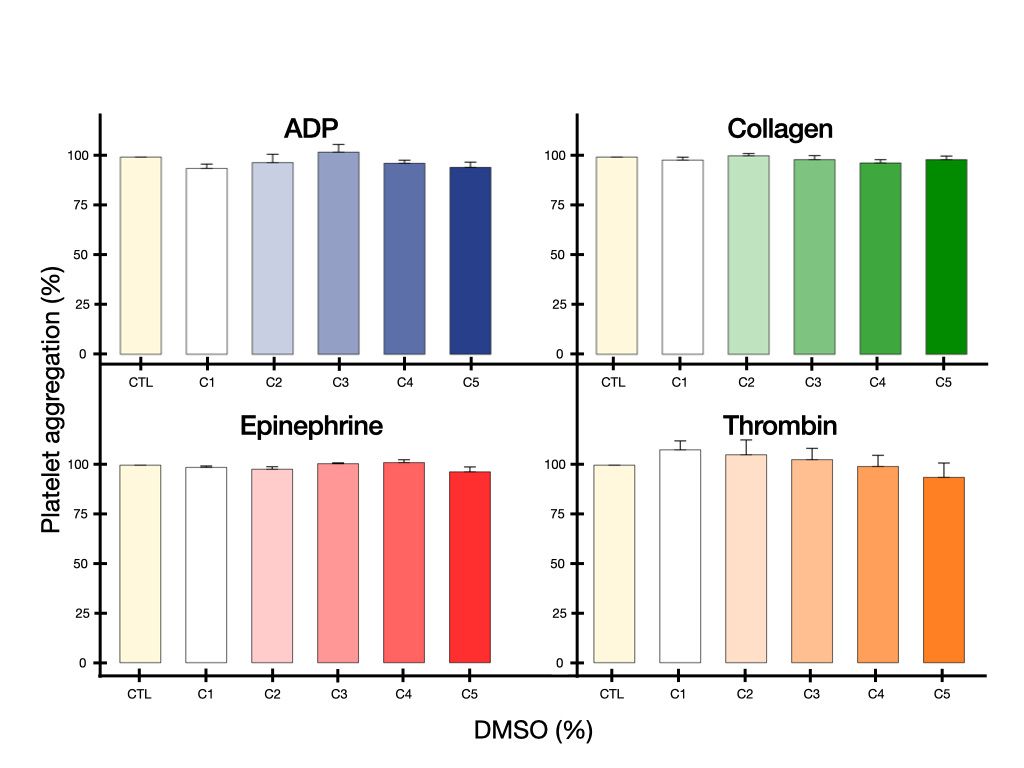

Supplement: Supplementary file 6 [file Image1.tif]

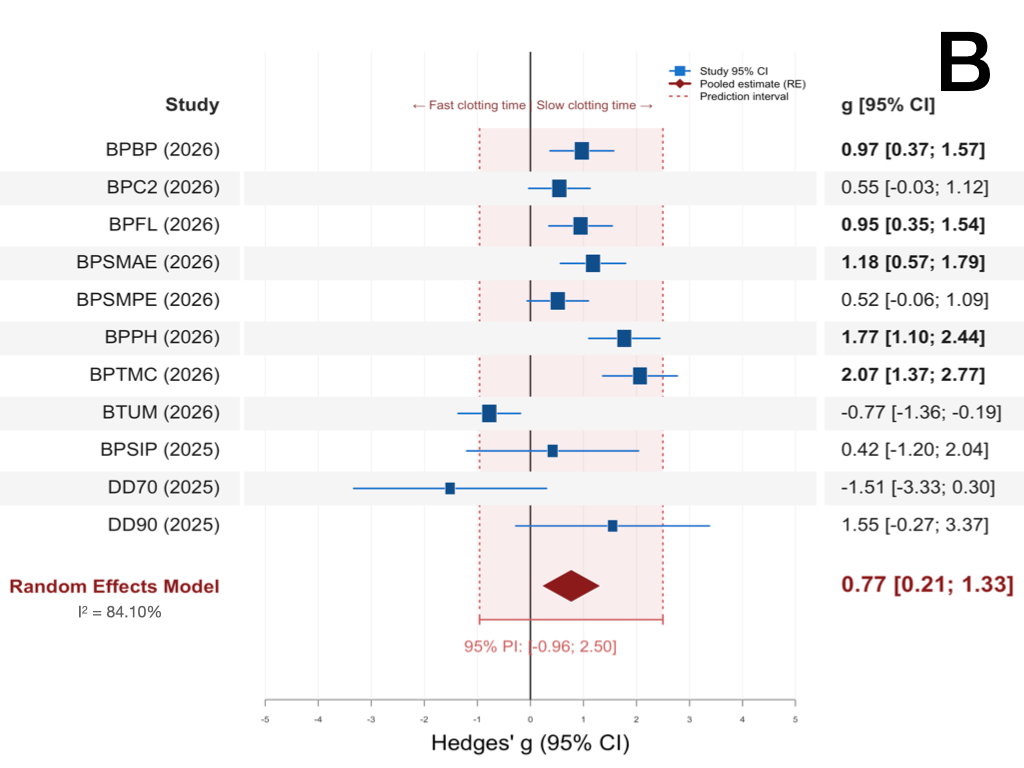

Supplement: Supplementary file 7 [file Image7.tif]

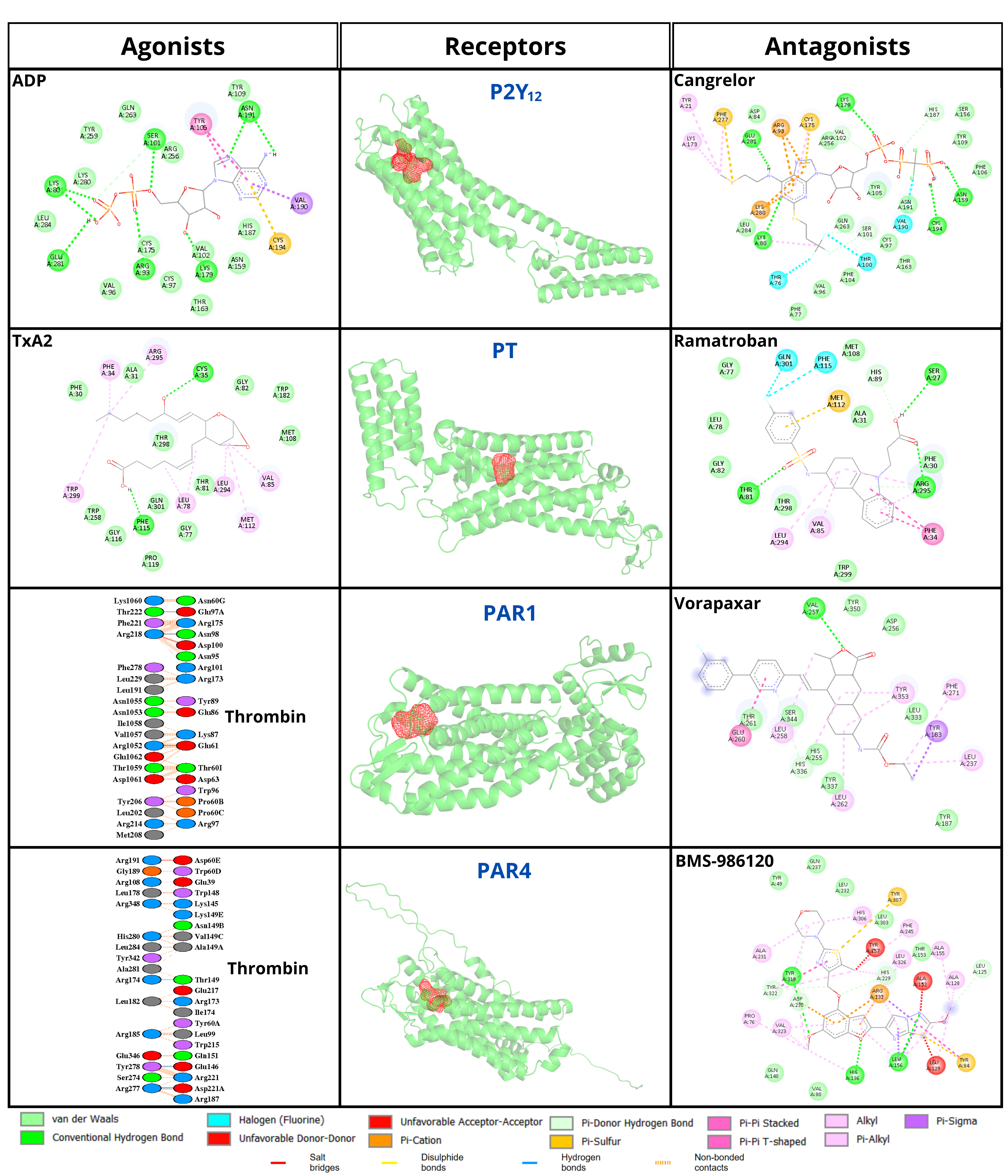

Supplement: Supplementary file 8 [file Image10.tiff]

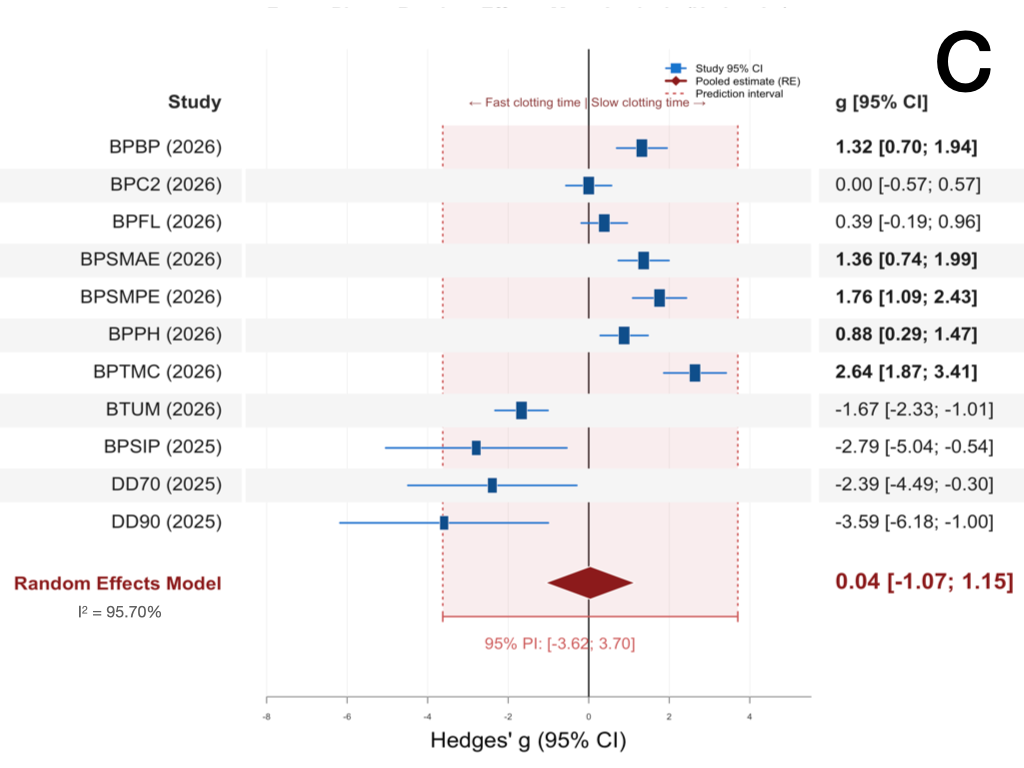

Supplement: Supplementary file 9 [file Image8.tif]

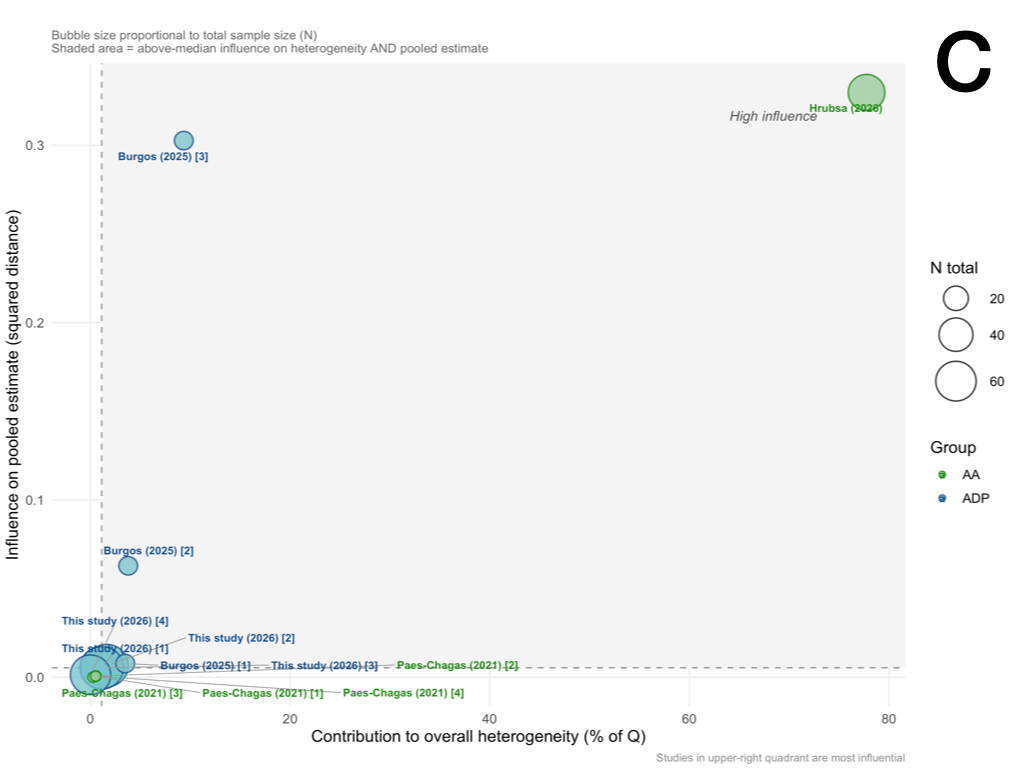

Supplement: Supplementary file 10 [file Image5.tif]
